# Thermal modulation of transcriptional states, biofilm architecture and survival strategies in *Cobetia marina*

**DOI:** 10.3389/fmicb.2026.1858040

**Published:** 2026-09-09

**Authors:** Claudia Ibacache-Quiroga, Oliver Schmachtenberg, Karoll González-Pizarro, Pablo Herrera, Felipe Tapia, Luna Osses-Lobos, Benjamin Serrano-Barba, Milan Stehlík, M. Alejandro Dinamarca

**Affiliations:** 1 Escuela de Nutrición y Dietética, Facultad de Farmacia, Universidad de Valparaíso, Valparaíso, Chile; 2 Centro de Micro-Bioinnovación (CMBi), Universidad de Valparaíso, Valparaíso, Chile; 3 Instituto de Biología, Facultad de Ciencias, Universidad de Valparaíso, Valparaíso, Chile; 4 Centro de Investigación y Gestión de Recursos Naturales (CIGREN), Universidad de Valparaíso, Valparaíso, Chile; 5 Centro Interdisciplinario de Neurociencia de Valparaíso, Universidad de Valparaíso, Valparaíso, Chile; 6 Escuela de Ingeniería Informática, Facultad de Ingeniería, Universidad de Valparaíso, Valparaíso, Chile; 7 Instituto de Estadística, Facultad de Ciencias, Universidad de Valparaíso, Valparaíso, Chile

**Keywords:** adaptive laboratory evolution, biofilm architecture, *Cobetia marina*, gene regulatory networks, marine bacteria, thermal adaptation

## Abstract

**Inroduction:**

*Cobetia marina* is a marine bacterium that sustains growth and biofilm formation across a broad thermal range, serving as an ideal model for exploring adaptability in thermally dynamic and warming oceans. In this study, we investigated how growth temperature reshapes transcriptional regulation and biofilm architecture in *C. marina* strain MM1IDA2H-1, as well as its trajectories under thermal stress.

**Methods:**

We integrated transcriptomic profiling with confocal and electron microscopy at different growth temperatures. Additionally, we conducted adaptive laboratory evolution (ALE) under progressive heat stress coupled with whole-genome sequencing.

**Results:**

Transcriptomes from cultures grown at 16 °C, 35 °C, 38 °C, and 41 °C were linked to physiology and biofilm structure, whereas ALE revealed strategies under warming stress. Low temperature promoted a biofilm-competent program driving motility and exopolysaccharide production. Conversely, growth at 41 °C induced a stress-survival state with repression of cooperative traits—quorum sensing—and the activation of DNA repair and oxidative stress responses. Exploratory network analyses predicted NarL, NtrC, CysB, and CsgD as putative components of a temperature-responsive control core, with a reduction in regulatory connectivity at 38 °C, representing a transitional stop-and-reprogram state. Finally, ALE selected for recurrent clone-specific mutations in *csgD1*, resolving a phenotypic trade-off by downregulating costly biofilm production to maintain growth capacity.

**Discussion:**

Overall, our findings show how *C. marina* transitions across a wide temperature range and under thermal stress via state-dependent network rewiring, offering a comprehensive eco-physiological framework that links environmental sensing, multicellular organization, and evolutionary trade-offs in highly dynamic and warming oceans.

## 1 Introduction

Bacteria are among the most ubiquitous, diverse, and evolutionarily successful forms of life on planet Earth. These microorganisms have persisted for nearly 3.8 billion years, and their long evolutionary history, together with their capacity to colonize virtually every known habitat, reflects a remarkable combination of ecological versatility, effective dispersal, and adaptive potential (Falkowski et al., 2008). Among these habitats, marine ecosystems offer a prime example of such environmental complexity, presenting highly diverse selective regimes and evolutionary pathways for microbial life (Fraschetti et al., 2008).

Marine habitats, including estuaries, sediments, coastal zones, intertidal pools, and hydrothermal vents, are characterized by pronounced variability in key environmental physicochemical parameters such as temperature, radiation, pH, salinity, and oxygen availability (Fraschetti et al., 2008). This spatial and temporal heterogeneity imposes strong selective pressures that shape microbial diversity, community succession, and biogeochemical processes across marine ecosystems (Stocker, 2012; Zhou et al., 2022). Among the physical and chemical variables, temperature is a key environmental factor governing the stratification and dynamics of the global ocean.

Seawater temperature is shaped by geological and oceanographic conditions, but it is primarily driven by climate and its associated multi-scale variability. An eleven-year study demonstrated that climate-driven variations in seawater temperature trigger pronounced seasonal successions in marine microbiome structure, with critical downstream implications for biogeochemical cycles (Larkin et al., 2025). Specifically, this high-resolution oceanographic record demonstrates that coastal and epipelagic waters experience strong seasonal thermal fluctuations; sea surface temperatures range from approximately 14 °C during cold, nutrient-rich winter upwelling regimes to over 24 °C during summer and autumn in temperate zones. In tropical regions, these baselines are elevated, with sea surface temperatures frequently exceeding 30 °C during El Niño-Southern Oscillation (ENSO) warming phases and extreme marine heatwaves (Hughes et al., 2017). These natural thermal fluctuations are superimposed upon a rapidly warming trend, as contemporary climate models and observational analyses confirm accelerating ocean warming rates in recent decades (Cheng et al., 2019). Therefore, the persistence and dynamics of bacterial communities in these warming habitats depend not only on dispersal but also on the cellular capacity to sense environmental shifts and, accordingly, adjust their ecophysiology. In this context, studies focusing on individual biological models provide a powerful framework for understanding how microbes respond and adapt evolutionarily to climate-driven thermal fluctuations.

The ubiquity of marine bacteria across dynamic oceanic environments arises from a complex interplay between dispersal and ecophysiological adaptability. Many taxa can transition between dormant, stress-resistant, and metabolically active states in response to changing environmental conditions, thereby maintaining survival and ecological function across contrasting niches (Morris et al., 2002; Müller et al., 2014; Sunagawa et al., 2015; Bradley et al., 2019; Wörmer et al., 2019; Hoarfrost et al., 2020; Boeuf et al., 2021; Clifton et al., 2024; Sadler et al., 2025). Such adaptive plasticity is especially evident among marine Pseudomonadota, one of the most abundant and widely distributed bacterial phyla in the oceans, whose members dominate important fractions of bacterioplankton communities and occupy habitats ranging from seawater and sediments to biofilms and host-associated environments (Delmont et al., 2018; Pollet et al., 2018; Zhou et al., 2020). A classic, ecologically critical example of this thermal responsiveness within the Pseudomonadota is represented by the genus *Vibrio*, where temperature upshifts have been shown to significantly enhance growth, accelerate resuscitation from viable-but-non-culturable (VBNC) states, and directly modulate pathogenicity and virulence traits (Sheikh et al., 2022). Consequently, evaluating how other, non-pathogenic eurythermal Pseudomonadota, such as members of the Halomonadaceae family, coordinate transitions between cooperative biofilm lifestyles and individual survival strategies under thermal variations or heat stress is crucial to understanding the evolutionary constraints and resilience of marine microbiomes in warming oceans.

Within this broad oceanographic framework, the family Halomonadaceae represents a particularly informative taxon. Members of the genus *Cobetia* exhibit remarkable ecological plasticity, allowing them to thrive across highly diverse marine environments (Moriya et al., 2020; Fernández-Juárez et al., 2022; Boyadzhieva et al., 2025). Specifically, *Cobetia marina* has emerged as a prominent marine model bacterium due to its robust capacity to form complex biofilms across different temperatures and pH levels (Yumoto et al., 2004; Fang et al., 2024; Stehlik et al., 2025), degrade petroleum-derived hydrocarbons (Ibacache-Quiroga et al., 2013) and synthesize high-value biomolecules, including exopolysaccharides, biosurfactants, and specialized enzymes such as extracellular phosphatases and phytases (Balabanova et al., 2024; Boyadzhieva et al., 2025). Comparative genomic and functional analyses suggest that this species is an ideal model for studying the molecular mechanisms underlying bacterial adaptation to environmental variability (Nedashkovskaya et al., 2024). Despite growing knowledge of its genomic repertoire and physiological diversity, the mechanistic basis by which *C. marina* integrates external cues into coordinated cellular responses remains poorly understood. Therefore, further studies are needed to clarify how gene regulatory systems link environmental sensing, cellular state transitions, and phenotypic outputs under variable environmental conditions found in marine habitats, and contribute to the ecological success of *C. marina*.

In this study, we implemented an integrated, multi-scale approach combining transcriptomic profiling (RNA-seq), three-dimensional confocal laser scanning microscopy (CLSM), and transmission electron microscopy (TEM), alongside adaptive laboratory evolution (ALE) coupled with whole-genome sequencing (WGS). Through this comprehensive framework, we aim to understand the physiological basis underlying the eurythermal nature of *Cobetia marina* strain MM1IDA2H-1 while simultaneously investigating its evolutionary responses to thermal stress.

## 2 Materials and methods

### 2.1 Bacterial strains, culture conditions, and baseline parameters

*Cobetia marina* strain MM1IDA2H-1 wild-type (WT) (CECT 7764) was routinely cultured in Marine Broth 2216 (Difco) under conditions approximating its halophilic and oligotrophic environment, as previously described (Ibacache-Quiroga et al., 2013). Cultivation was performed either in an orbital shaking incubator at 100 rpm or in a temperature-controlled spectrophotometer equipped with incubation and shaking capacity (Tecan, Infinite 200 Pro).

### 2.2 Transcriptomic profiling and co-expression analysis

Cultures were grown in 96-well plates with marine broth to mid-exponential phase at 16 °C, 35 °C, 38 °C, and 41 °C. These temperatures were selected based on the growth profile and biofilm formation, with respect to the optimal growth condition at 35 °C (Stehlik et al., 2025). To establish a robust biological replication structure, each temperature condition was evaluated using three independent 96-well culture plates (biological replicates, *n* = 3). Within each independent 96-well plate, 93 wells were inoculated with a 16-h *C. marina* strain MM1IDA2H-1 starter culture grown at 35 °C in marine broth, while the remaining three wells were left uninoculated as sterile controls. To harvest sufficient biomass for high-quality RNA extraction and assess biological diversity among intra-plate populations, the contents of all 93 wells within a single culture plate were pooled into a single experimental unit for RNA extraction. Total RNA was extracted using the RNeasy Mini Kit (Qiagen). Before downstream library preparation, automated capillary electrophoresis was executed on an Agilent TapeStation 4150 system (Agilent Technologies) to evaluate total RNA concentration and integrity. All twelve experimental samples met standardization criteria, exhibiting a concentration ≥ 50 ng/μL and an RNA Integrity Number (RIN) ranging between 6.5 and 8.8 (mean RIN^e^ = 8.25), confirming optimal transcript stability (Supplementary Table S1). Library preparation and RNA sequencing were performed by SeqCenter (Pittsburgh, PA, United States). Samples were DNase-treated with Invitrogen DNase (RNase-free). Library preparation was performed using Illumina’s Stranded Total RNA Prep Ligation with Ribo-Zero Plus kit and 10 bp unique dual indices (UDI). Sequencing was done on a NovaSeq X Plus (Illumina), producing paired-end 150 bp reads. Demultiplexing, quality control, and adapter trimming were performed with bcl-convert (v. 4.2.4). Transcript quantification was carried out with salmon (v. 1.11.4) (Patro et al., 2017), using the closed genome of *C. marina* MM1IDA2H-1 (GCF_002916775.2) as the reference. Differential gene expression was analyzed with the *DESeq2* package (v.1.50.2) (Love et al., 2014) in R (v.4.5.2) (R Core Team, 2024), using 35 °C as the control condition. Raw transcript-level abundances were imported from Salmon quantification files using the tximport() function, which summarized transcript-level estimates into gene-level counts using a custom transcript-to-gene mapping generated from the reference annotation. A DESeq2 dataset was constructed from the imported count matrix using DESeqDataSetFromTximport(), and raw counts were normalized using the DESeq2 median-of-ratios method through sample-specific size factor estimation implemented in the estimateSizeFactors() step within the DESeq() workflow. Gene-wise dispersion estimates were calculated using the DESeq2 negative binomial model with the estimateDispersions() procedure, in which dispersions were estimated from biological replicates, fitted to a mean–dispersion trend, and then shrunk toward the fitted trend. Differential expression analysis was performed using the DESeq() function, which applies the negative binomial Wald test (nbinomWaldTest()) for statistical inference. Results were extracted using the results() function, with multiple testing correction performed using the Benjamini–Hochberg false discovery rate method (pAdjustMethod = “BH”). Genes were considered differentially expressed when the adjusted *p*-value (padj) was <0.05 and the absolute log2 fold change (|log2FoldChange|) was ≥1. Variance-stabilized expression values were generated using vst() and rlogTransformation() for downstream visualization and clustering analyses.

A weighted gene co-expression network analysis (WGCNA) was performed to identify gene expression modules associated with experimental conditions and phenotypic traits using the *WGCNA* package (v.1.73) (Langfelder and Horvath, 2008) in R (v.4.5.2). Gene expression values obtained from Salmon quantification were imported into DESeq2 using tximport and normalized using DESeq2 size factors. A variance-stabilizing transformation (VST) was applied to reduce the dependence between variance and mean expression before network construction. After quality control using the goodSamplesGenes function implemented in WGCNA, all 12 samples and 3,418 genes were retained for downstream analysis. The soft-thresholding power was selected using the pickSoftThreshold function by evaluating powers ranging from 1 to 20. A soft-thresholding power of *β* = 1 was selected and used for network construction. The co-expression network was constructed using an unsigned topological overlap matrix (TOM) with Pearson’s correlation as the similarity measure. Modules were detected using the blockwiseModules function with a minimum module size of 30 genes and a module merging threshold of 0.25. Module eigengenes were calculated as the first principal component of each module and were correlated with experimental traits using Pearson correlations. Statistical significance of module–trait associations was assessed using Student’s asymptotic test, and *p*-values were corrected for multiple testing using the Benjamini–Hochberg false discovery rate (FDR) method. Significant module–trait associations were considered at FDR < 0.05.

### 2.3 Transcriptome annotation and functional overrepresentation analysis

Annotation of the obtained transcriptomes was performed using two main approaches: GenBank and *Kyoto Encyclopedia of Genes and Genomes* (KEGG) pathways (Kanehisa et al., 2024). For GenBank annotation, *Cobetia marina* strain MM1IDA2H-1 closed genome (GCF_002916775.2) was used as reference, and transcriptomes were annotated according to “Locus tag” identifier using dplyr package in R (v.4.5.2). For the enrichment analysis of KEGG pathways, *C. marina* strain MM1IDA2H-1 genome was annotated using BlastKOALA online tool^1^ (Kanehisa et al., 2016), and transcriptomes were annotated as described above.

The enrichment analysis of KEGG pathways in the bacterial transcriptome at different temperatures (16 °C, 38 °C, and 41 °C) was performed with the *clusterProfiler* (v. 4.16.0) (Wu et al., 2021), *enrichplot* (v. 1.28.4) (Yu, 2026), *KEGGREST* (v. 1.48.1)(Tenenbaum and Maintainer, 2025) packages in R (v. 4.5.1). Functional annotations of *C. marina* strain MM1IDA2H-1 genes were generated with eggNOG-mapper (Huerta-Cepas et al., 2019; Cantalapiedra et al., 2021). KEGG Orthology (KO) identifiers were extracted and standardized to generate a gene-KO correspondence table. KO-to-pathway associations were subsequently retrieved from the eggNOG annotations, and a custom gene-to-KEGG pathway database was constructed for downstream enrichment analyses. To reduce biases arising from differences in functional annotation coverage across comparisons, the background universe was defined as the intersection of all genes with KEGG pathway annotations present in all three experimental conditions. This common background set was used in all enrichment analyses. KEGG pathway overrepresentation analysis was performed using the compareCluster() function from the clusterProfile package. The minimal and maximal gene set sizes were set at 3 and 500, respectively. Enrichment analyses were conducted separately for upregulated, downregulated, and the complete set of differentially expressed genes. Statistical significance was assessed using the hypergeometric test implemented in the enricher() function, and *p*-values were adjusted for multiple testing using the Benjamini–Hochberg procedure. Enriched pathways were considered significant at FDR ≤ 0.05. For each pathway, the number of associated genes, GeneRatio, BgRatio, and enrichment factor were calculated. The enrichment factor was defined as the ratio between the observed gene proportion in the query set and the expected gene proportion in the background universe. Additionally, for the visualization of differentially expressed genes, the following features and pathways were selected for the biological context analysis: quorum sensing (map02024); biofilm (map02025, map02026, map05111); flagellar assembly and bacterial chemotaxis (map02030, map02040); transcription (map03020, map03022; map03040), translation (map03008, map03010, map03013; map03015; map00970), folding sorting and degradation (map03018, map03050, map03060, map04141, map04120, map04122, map04130), and replication and repair (map03030, map03410, map03420, map03430, map03440, map03450, map03460). Selected pathways databases were manually downloaded from the KEGG Application Programming Interface (API) for DEG visualization.

### 2.4 Confocal laser scanning microscopy and three-dimensional biofilm analysis

Biofilm architecture and bacterial viability were analyzed using confocal laser scanning microscopy (CLSM). Biofilms were obtained by immersing sterile glass slides in a Petri dish containing 15 mL of Marine Broth 2216 inoculated with *Cobetia marina* strain MM1IDA2H-1. Samples were incubated for 28–72 h at different temperatures without agitation. Slides were recovered and washed with sterile NaCl solution (0.9% w/v), air-dried, and stained with Live/Dead™ BacLight™ (Invitrogen) according to the manufacturer’s instructions. Images were obtained using a spectral confocal microscope (Stellaris 5, Leica), using the 40x/1.3 oil immersion objective (600 Hz bidirectional scan, 64.5 μm pinhole, 2.5x zoom, 488 nm and 561 nm stimulation and 500 nm–540 nm, 600 nm–651 nm detection ranges, respectively, 0.5 μm z-scale). For biofilm depth analysis, a total of 10 regions of interest (ROIs), each measuring 116.07 × 116.07 μm, were selected from one slide for each temperature condition (16 °C, 35 °C, 41 °C). The depth along the z-axis was determined by the biofilm depth within the ROI. The volumes of live and dead bacteria are expressed as the volume per ROI cube. Volumes were calculated using a custom script written in Python 3.12.4 (available at https://github.com/FTapiaP/Cobetia3D/). Briefly, images were thresholded and blobs representing bacteria were separated by morphology and summed up using a binary mask. Three-dimensional renders were obtained using the PyVista (v. 0.46.5) library. Data were tested for normality and compared using either one-way ANOVA with Welch’s correction for inequality of variance followed by Games-Howell *post hoc* tests for normal variables or Kruskal-Wallis followed by Dwass-Steel-Critchlow-Fligner post hoc tests otherwise. Data are expressed as mean ± standard deviation; *p*-values were considered significant when *α* < 0.05 and are presented for the significant post hoc test.

### 2.5 Transmission electron microscopy

Biofilms subjected to different temperatures were prepared using sterile agarose slides (0.8% w/v) immersed in sterile Petri dishes containing 15 mL of Marine Broth 2216 inoculated with *Cobetia marina* strain MM1IDA2H-1. Samples were incubated for 48–72 h at the selected temperatures. Portions of the agarose samples containing bacterial biofilm were fixed for 2 h at room temperature in Karnovsky’s solution (4% paraformaldehyde and 2.5% glutaraldehyde) buffered with 0.1 M sodium cacodylate buffer (pH 7.2) containing 4% sucrose. Subsequently, the samples were washed in the same buffer and post-fixed with 1% osmium tetroxide in 0.5% potassium ferrocyanide for 1 h at room temperature. The biofilm samples were dehydrated through a graded ethanol series, with acetone used as an intermediate solvent, and subsequently embedded in epoxy resin (Epon, Embed-812 EMS). Polymerization was carried out in an oven at 60 °C for 72 h. Ultrathin sections (60–90 nm) were obtained using a PowerTome XL ultramicrotome (RMC Boeckeler). The sections were contrasted with uranyl acetate and lead citrate and mounted on 200-mesh copper grids. Images were taken using a JEOL JEM-1400 Flash transmission electron microscope operated at an accelerating voltage of 80 kV.

### 2.6 Detection of curli-like structures

To identify curli-like structures in *Cobetia marina* strain MM1IDA2H-1 growing at different temperatures, Congo Red (CR) staining was used. For this purpose, strain MM1IDA2H-1 was inoculated on Marine agar 2216 (Difco) supplemented with CR at a final concentration of 800 μg/mL (Xiong et al., 2022). Plates were incubated at 16 °C, 35 °C, 38 °C, and 41 °C for 48–72 h, and colonies were visualized using a Nikon SMZ1500 stereomicroscope.

### 2.7 Adaptive laboratory evolution (ALE) under thermal stress

To investigate *Cobetia marina* strain MM1IDA2H-1’s adaptation to thermal stress, an adaptive laboratory evolution (ALE) experiment was designed, adapted from a previous evolution study by Roemhild et al. (2015). Bacterial evolution was performed in 96-well plates in Marine Broth 2216 culture medium at progressively increasing temperatures starting from the optimal growth temperature (35 °C). Plates were incubated in a spectrophotometer equipped with incubation mode and temperature control (Tecan Infinite 200 Pro) at 2 rpm linear agitation. Briefly, 93 wells were inoculated with 10^6^ cells of *Cobetia marina* strain MM1IDA2H-1 from a 16-h culture, while three wells served as a negative control without inoculation; all were incubated at 35 °C until mid-exponential phase. Then, 1 μL of culture from each well was inoculated into a new plate containing fresh medium, and the temperature was increased by 0.5 °C. The evolutionary process was carried out up to 42 °C. Optical density at 600 nm (OD_600nm_) was continuously registered every 15 min, and biofilm formation was quantified at the final step of each temperature. Clones from replicates showing growth were isolated on marine agar and freeze-preserved for further characterization.

### 2.8 Growth kinetics and biofilm formation assays

Planktonic growth kinetics of each step of the ALE experiment were continuously monitored by measuring the optical density at 600 nm (OD_600_) of each 93 linages every 15 min using a temperature-controlled spectrophotometer (Tecan, Infinite 200 Pro) with agitation. From the resulting OD_600_ data, the maximum specific growth rate (μ_max_) for each of the 93 lineages at every thermal step was calculated by determining the maximum slope of the natural logarithm of OD_600_ versus time during the exponential growth phase, using the *growthrates* package (v. 0.8.5) (Hall et al., 2014) in R.

Biofilm formation was assessed using the standardized 96-well crystal violet assay originally described by O’Toole (O'Toole, 2011). As described in 2.6, ALE cultures were grown in Marine Broth 2216 at each temperature until mid-exponential phase. Following incubation, planktonic cells were removed, and wells were washed three times with sterile 0.9% (w/v) NaCl solution. Plates were air-dried and stained with 0.1% (w/v) crystal violet for 15 min. Afterward, plates were rinsed thoroughly, air-dried, and the adhered dye was solubilized with ethanol: acetone (70:30). Crystal violet absorbance was measured at 540 nm, and biofilm formation was normalized to planktonic biomass measured at OD_600_, expressed as the OD_540_/OD_600_ ratio.

### 2.9 Genomic DNA extraction, whole-genome sequencing (WGS), and variant calling

DNA extraction from all selected strains was performed using the DNeasy Blood & Tissue kit according to the manufacturer’s instructions (Qiagen). DNA integrity and size were evaluated using miniaturized electrophoresis on a TapeStation 4,150 (Agilent Technologies) with Genomic DNA Reagents and Genomic DNA ScreenTape (Agilent Technologies). Nanopore sequencing libraries were prepared using the Native Barcoding Kit (Oxford Nanopore Technologies, ONT) according to the manufacturer’s instructions and sequenced on a GridION platform (ONT). Sequence basecalling was performed in the MinKNOW (v. 25.05.14) platform using standard parameters, and only sequences with Qscore > 12 were selected for further studies.

For variant calling, reads were aligned with the reference genome (GCA_002916775.2) using Minimap2 (v. 2.26) (Li, 2018). SAM files were converted to BAM files using Samtools (v.1.20) (Danecek et al., 2021). Variant calling was performed using Clair3 (v. 1.2.0) following the pipeline described by Yu et al. (2023). Called variants were filtered based on QUAL >30, frequency >0.6, and depth >40x. Exceptionally, reads with depth frequency >0.9 and depth <40x were kept for further analysis. The potential impact of the sequencing method on variant calling was assessed by sequencing the wild-type strain as a blank, given differences from the reference genome (Illumina-ONT combined sequencing strategy).

### 2.10 Regulatory network reconstruction

To identify potential transcription factors, the genome of the *Cobetia marina* strain MM1IDA2H-1 was analyzed using predictTF (Oliveira Monteiro et al., 2022). Analysis of position weight matrices of motifs associated with the identified transcription factors in genes´ upstream regions (350 bp) was performed online in the Prodoric database webpage^2^ (Münch et al., 2003), using its integrated *Virtual Footprint* tool for regulon analysis, and the *C. marina* strain MM1IDA2H-1 genome was directly uploaded from its GenBank search tool.

### 2.11 Quantitative real-time PCR (qRT-PCR) validation

To experimentally validate the transcriptomic profiles from the RNA-seq analysis, the expression levels of selected target genes were quantified by qRT-PCR on the same RNA samples. The reactions were performed using the Brilliant III Ultra-Fast SYBR Green qRT-PCR Master Mix (Agilent Technologies) on an Agilent Mx3000P real-time PCR system. A subset of genes representing diverse functional categories was selected, including *dnaK*, *recA*, cold-shock protein, polysaccharide export protein, and the transcriptional regulators *csgD1*, MarR, and LysR. Primer sequences are detailed in Supplementary Table S2. The thermocycling protocol consisted of an initial reverse transcription step at 50 °C for 10 min, an initial denaturation at 95 °C for 3 min, and 40 amplification cycles comprising denaturation at 95 °C for 5 s, annealing at 60 °C for 20 s, and extension at 72 °C for 15 s. A final dissociation curve analysis was performed to confirm amplicon specificity. Relative gene expression across the thermal gradient (16 °C, 38 °C, and 41 °C) was calculated using the 2^−ΔΔCt^ method, with the *rpoD* gene as the internal reference and the 35 °C optimal growth condition as the baseline calibrator. Statistical analysis was conducted in R using the emmeans package (v. 2.0.3). Expression differences were evaluated by fitting linear models to the ΔCt values (ΔCt ~ group + replicate), followed by Dunnett’s multiple-comparison test against the control group. Statistical significance was defined using adjusted *p*-values.

### 2.12 Experimental design and statistical analysis

High-throughput cultures and phenotypic evaluations were performed in 96-well plate formats. Depending on the specific assay, the internal wells were either analyzed individually (e.g., biofilm quantification, ALE phenotypic trajectories) or completely pooled per plate to obtain sufficient biomass representing a single biological replicate (e.g., RNA extraction for RNA-seq and qRT-PCR).

Differential gene expression was assessed using negative binomial Wald tests, with *p*-values adjusted for multiple comparisons using the Benjamini–Hochberg false discovery rate (FDR) procedure. Genes were considered differentially expressed when the adjusted *p*-value (padj) was < 0.05 and the |log2 fold change| ≥ 1. For gene co-expression network análisis (WGCNA), the significance of module–trait associations was evaluated using Student’s asymptotic test, and *p*-values were corrected using the Benjamini–Hochberg FDR method; associations were considered significant at FDR < 0.05. Functional enrichment analyses were evaluated using hypergeometric tests, with multiple-testing correction performed using the Benjamini–Hochberg procedure, and enriched pathways were considered significant at FDR ≤ 0.05. For qRT-PCR validation, expression differences were analyzed using linear models fitted to ΔCt values, followed by Dunnett’s multiple-comparison test against the control group. Statistical significance was determined using adjusted p-values, with a significance threshold of *p*-value <0.05. For Confocal laser scanning microscopy, data were tested for normality and compared using either one-way ANOVA with Welch’s correction for inequality of variance followed by Games-Howell *post hoc* tests for normal variables or Kruskal-Wallis followed by Dwass-Steel-Critchlow-Fligner *post hoc* tests otherwise. Data are expressed as mean ± standard deviation, *p*-values were considered significant when *α* < 0.05, and are presented for the significant post hoc test.

For phenotypic characterization in ALE, differences in μ_max_ and biofilm formation among temperature conditions were assessed using Dunn’s multiple-comparison test, with the 35 °C condition used as the reference. *p*-values were adjusted for multiple comparisons using the Benjamini–Hochberg procedure. Statistical significance was determined using adjusted *p*-values, with a significance threshold of *p*-value <0.05.

### 2.13 Data availability

The datasets presented in this study are available in online repositories. Raw RNA sequencing data are available in the NCBI Gene Expression Omnibus (GEO) repository under BioProject accession number GSE282360. Whole-genome sequencing data from the evolved derivative clones were deposited in the NCBI Sequence Read Archive (SRA) under BioProject PRJNA1453919 (BioSamples: SAMN57277002 through SAMN57277009). The custom Python scripts utilized for three-dimensional confocal image processing and volumetric analysis, along with the R pipelines for transcriptomic data, are openly accessible in the GitHub repository at https://github.com/FTapiaP/Cobetia3D/

## 3 Results

### 3.1 Temperature modulates the transcriptional program of *Cobetia marina* strain MM1IDA2H-1 modifying gene expression associated with social behavior, metabolism, and genomic maintenance

*Cobetia marina* strain MM1IDA2H-1 grows within a broad temperature range (Supplementary Figure S1), from 0 to 41 °C, with the optimal *in vitro* growth temperature being 35 °C (Stehlik et al., 2025). Therefore, to assess its temperature-dependent transcriptomic response, four key growth temperatures (16 °C, 35 °C, 38 °C, and 41 °C) were selected according to different phenotypes, including biomass production, maximum growth rate (μ_max_), growth kinetics, and biofilm formation. For this purpose, non-ribosomal RNA from bacteria exposed to the selected temperatures was sequenced, and differentially expressed genes (DEGs) were quantified and compared to the optimal growth condition (35 °C). RNA, sequencing statistics, and quality control information are detailed in Supplementary Table S1. Briefly, a total of 276.86 million raw reads and 70.74 G clean bases (93.39%) were obtained in this study.

**Figure 1:**
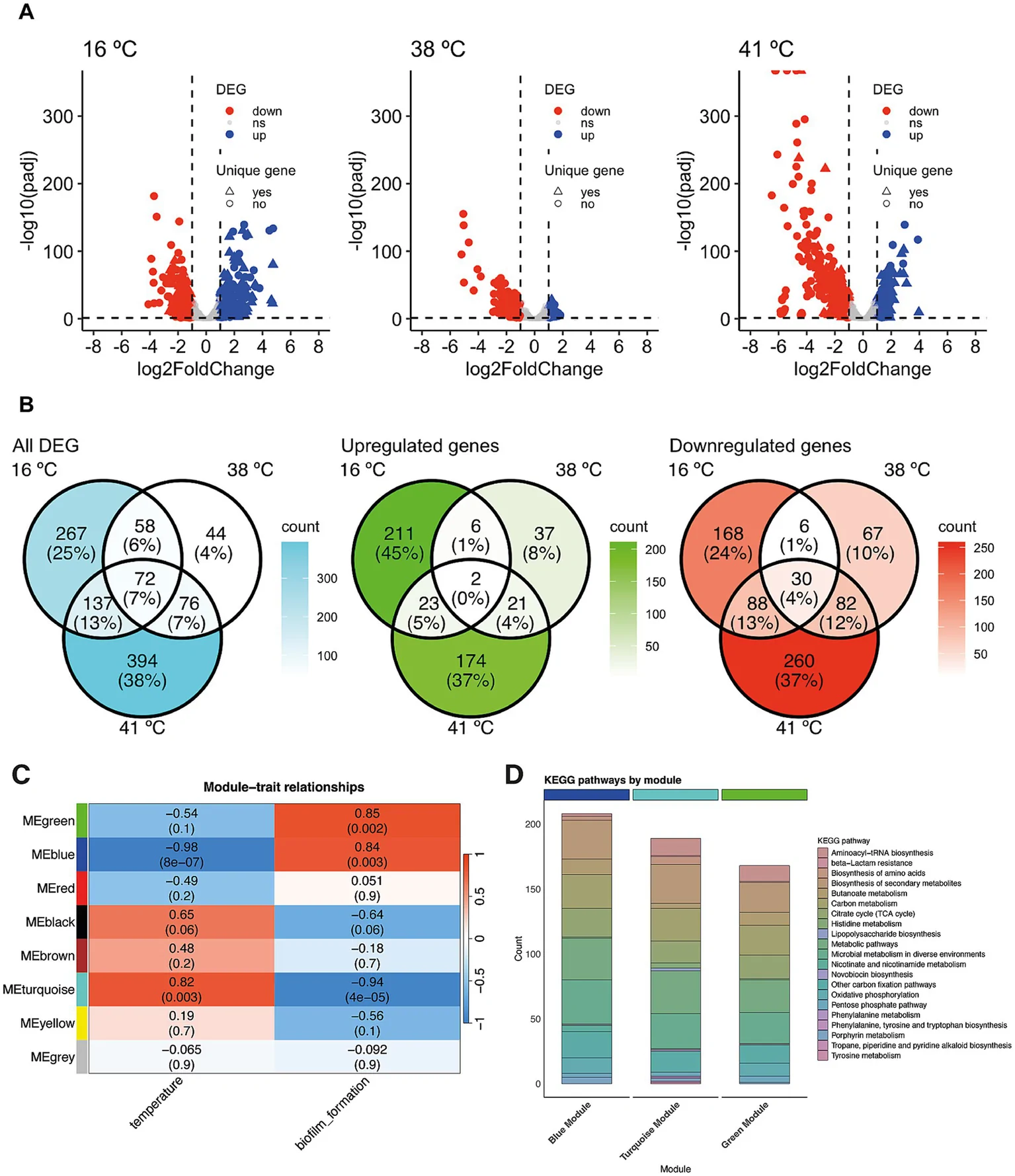
Gene expression analysis of *Cobetia marina* MM1IDA2H-1 exposed to different temperatures. **(A)** Differential gene expression at 16 °C, 38 °C, and 41 °C with respect to 35 °C. Differentially expressed genes (DEGs) were defined utilizing an absolute threshold of |*log2FoldChange*| ≥ 1 and a Benjamini-Hochberg adjusted *p*-value (*padj*) < 0.05. **(B)** Venn diagrams of differentially expressed genes (DEG) at the evaluated temperatures. **(C)** Weighted Gene Co-expression Network Analysis (WGCNA) analysis of *C. marina* MM1IDA2H-1 under temperature stress and its association with biofilm formation. Statistical significance of module–trait associations was assessed using Student’s asymptotic test, and *p*-values were corrected for multiple testing using the Benjamini–Hochberg false discovery rate (*FDR*) method. Significant module–trait associations were considered at *FDR* < 0.05. **(D)** KEGG annotation of WGCNA modules associated with temperature and biofilm formation.

According to the results, the condition at which *C. marina* strain MM1IDA2H-1 showed the highest number of DGEs (|log2FoldChange| ≥ 1, padj <0.05) was at 41 °C (679), followed by 16 °C (533), while at 38 °C this bacterium presented the lowest number of DEGs (246) (Figure 1A) (Supplementary Tables S3–S5). As shown in Figure 1B, the three temperature conditions shared a common core of 72 DEGs. In addition, 267, 44, and 394 genes were differentially expressed exclusively at 16 °C, 38 °C, and 41 °C, respectively. Analysis of shared upregulated and downregulated genes among all conditions revealed a common regulatory response at 16 °C and 41 °C, the two extreme temperatures of this study. These conditions shared 25 differentially upregulated and 118 downregulated genes.

To complement the differential expression profiles, gene co-expression patterns were analyzed in *C. marina* strain MM1IDA2H-1 in response to growth temperature. Weighted Gene Co-expression Network Analysis (WGCNA) identified eight discrete co-expression modules from the global transcriptomic data (Figure 1C). When evaluating the relationship between these modules and growth temperature, the results showed a negative correlation with the blue module and a positive correlation with the turquoise module. Concurrently, the relationship between these modules and biofilm formation, a phenotypic trait previously characterized in this microorganism that is affected by temperature, was analyzed. In contrast to the thermal trend, biofilm formation capacity showed a positive correlation with the green and blue modules and a negative correlation with the turquoise module. The overall composition of each module was annotated using the KEGG database and shown in Figure 1D.

**Figure 2:**
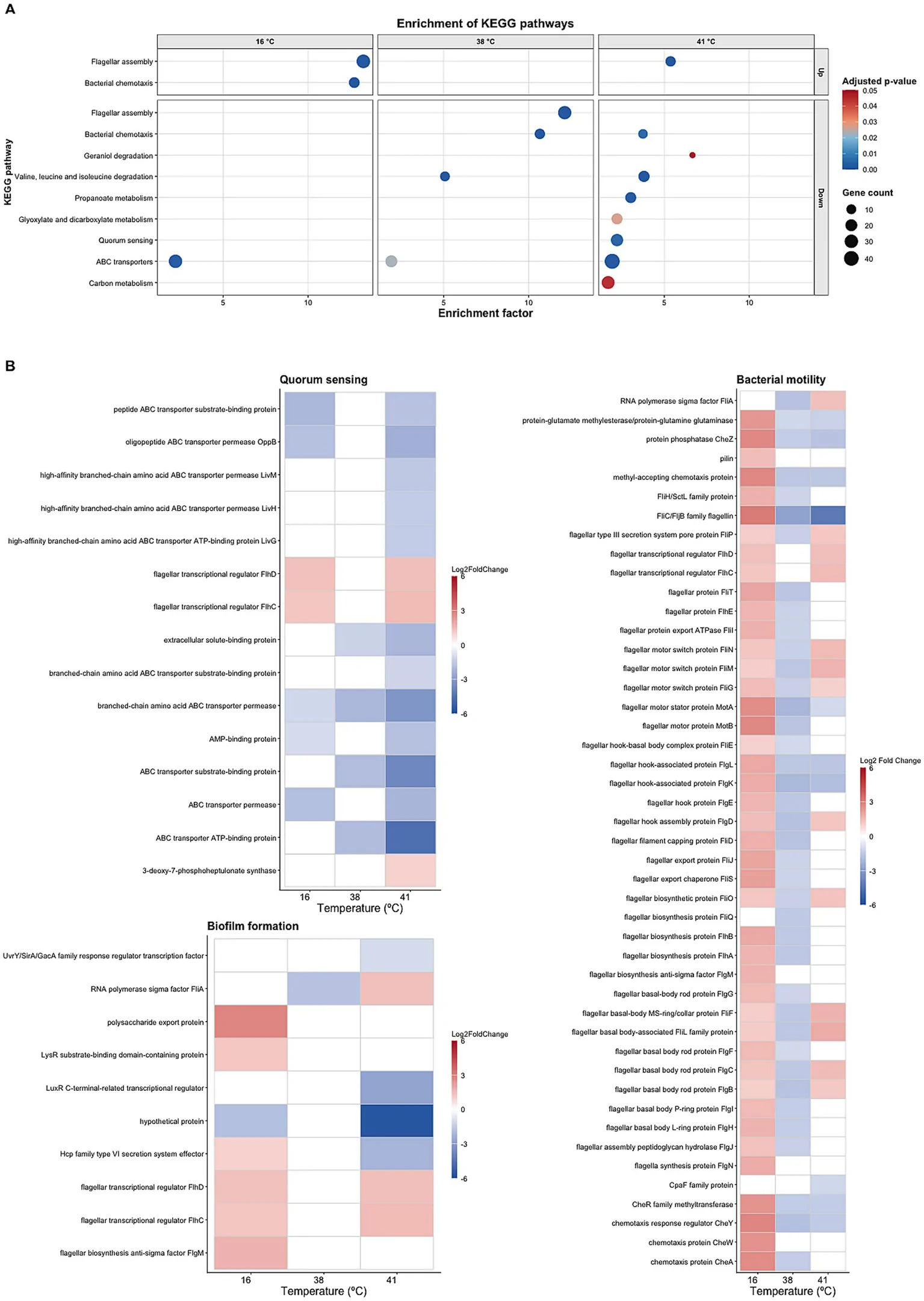
Bioinformatic screening and targeted transcriptional profiles of thermally modulated KEGG pathways in *Cobetia marina* MM1IDA2H-1. **(A)** KEGG pathway enrichment analysis displaying significantly upregulated (Up) and downregulated (Down) functional categories across a thermal gradient (16 °C, 38 °C, and 41 °C); circle size represents gene count, and color intensity indicates the statistical significance (adjusted *p*-value). Statistical significance was assessed using the enricher() function, and *p*-values were adjusted for multiple testing using the Benjamini–Hochberg procedure. Enriched pathways were considered significant at FDR ≤ 0.05. **(B)** Detailed expression profiles and sub-network analysis of selected quorum-sensing, motility, and biofilm-formation genes.

Additionally, to understand the functional implications of the transcriptomic shift observed at different temperatures, KEGG pathway enrichment analysis was performed. As shown in Figure 2A, when analyzing upregulated genes, exposure to 16 °C significantly enriched the expression of flagellar assembly and chemotaxis pathway genes. As the temperature increases to 38 °C, the expression pattern undergoes a drastic inversion, marked by the significant downregulation of the afore mentioned flagellar and chemotaxis machineries. Under thermal stress (41 °C), this repression extends to communication and energy-yielding processes. Notably, the Quorum-sensing pathway is significantly repressed, along with broad metabolic shutdowns, including carbon metabolism and specific amino acid degradation pathways. At the othe hand, the flagellar assembly pathway is enriched at this temperature. In summary, this enrichment analysis, in which motility is favored at low temperatures and high temperatures, and quorum sensing and energetic metabolism are repressed under severe heat stress, provides a robust data-driven biological context. Guided by these enrichment patterns, these specific categories provided the biological rationale for analyzing individual gene expression dynamics in downstream analyses (Figure 2B).

As shown in Figure 2B, the transcriptomic profile at 16 °C is dominated by the coordinated upregulation of pathways governing environmental exploration and community behavior. The Bacterial motility module exhibits a massive induction of flagellar assembly components (e.g., *flg*, *fli*, and *flh* gene families) and chemotaxis machinery. Concurrently, elements within the Biofilm formation and Quorum sensing modules are highly expressed. Additionally, several strongly upregulated genes belong to mobile genetic element/phage-associated functions, including a major capsid protein (log2FC ≈ +4.75) and additional phage structural/processing proteins (e.g., head decoration and head maturation protease; log2FC ≈ +3.50) (Supplementary Table S3, Figure S2). Additionally, 16 °C induced stress adaptation and central housekeeping components that often track physiological transitions, such as genes encoding the ferric uptake regulator (Fur) (log2FC ≈ +1.59) and the uracil/thymine-sensing master regulator (RutR) (log2FC ≈ +1.28). At 16 °C, direct biofilm-associated gene induction encompasses coordinated upregulation of exopolysaccharide biosynthesis and export (polysaccharide biosynthesis tyrosine autokinase, polysaccharide export protein), pilin, flagellar, OMVs/OMPs and chemotaxis machinery (*fli*/*flg*/*mot*/*che* loci/*cheR*), and a phage structural gene module (Supplementary Figure S2). This distinct transcriptional switch provides a basis for hypothesizing that lower temperatures promote a socially interactive, biofilm-competent state.

Consistent with the predominantly repressive transcriptional profile observed at this intermediate temperature, growth at 38 °C was characterized by strong downregulation of genes involved in iron acquisition and outer membrane transport (Supplementary Table S4). Among the most strongly repressed loci were multiple TonB-dependent siderophore receptors and associated iron uptake components, including a TonB-dependent receptor (log2FC ≈ −5.07) and an iron–siderophore ABC transporter substrate-binding protein (log2FC ≈ −5.04), together with their corresponding permease and ATP-binding subunits. This repression of iron acquisition and envelope-associated uptake systems suggests that growth at 38 °C triggers an early “redirection” of membrane and transport efforts compared to 35 °C. Two canonical chaperone genes—GroES (log2FC ≈ +1.51) and GroEL (log2FC ≈ +1.53) are induced, consistent with heightened protein-folding demand at elevated temperature.

At 41 °C, *C. marina* strain MM1IDA2H-1 exhibited strong repression of genes encoding transport and uptake functions, including a TRAP-type transporter substrate-binding protein (log2FC ≈ −4.74) and TonB-dependent iron acquisition systems (log2FC ≈ −6.20 and −6.25) (Supplementary Table S5). Concurrently, genes associated with stress adaptation and core cellular maintenance were upregulated, notably a sugar porter family MFS transporter, chemotaxis control CheR, and several redox-related enzymes. Under these conditions, the transcriptional program includes upregulation of DEAD/DEAH box helicase, RecO, DNA polymerase IV, DNA-formamidopyrimidine, catalase, multiple oxidoreductases, and Hsp90-family chaperone HtpG. Additionally, genes involved in capsular polysaccharide biosynthesis (UDP-Glucose Dehydrogenase) and polysaccharide export are induced at these temperatures.

### 3.2 Experimental validation of temperature-dependent transcriptomic profiles

To robustly validate the reliability of the global RNA-seq differential expression analysis, qRT-PCR was conducted on a subset of seven genes representing key functional categories altered by thermal condition: protein homeostasis and DNA repair (*dnaK*, *recA*, cold-shock protein), biofilm and envelope structural components (polysaccharide export protein), and transcriptional regulation (*csgD1*, MarR, and LysR families). As shown in Supplementary Figure S3, the qRT-PCR expression patterns were highly consistent with the transcriptomic data. For instance, the gene encoding cold-shock protein was significantly upregulated at 16 °C, with a Fold Change (FC) of +2.81 (*p*-value = 0.02). Despite not reaching statistical significance, a slight increased expression was observed in the genes encoding the polysaccharide export protein (BOH68_RS11430) and MarR family transcripotional regulator (BOH68_RS09310), with FCs of +1.83 (*p*-value = 0.06) and +1.3 (*p*-value = 0.33), respectively, concordant with RNAseq results. Consistent with the transcriptomic repression of biofilm-related pathways under extreme heat stress, the expression of the master regulator *csgD1* (FC = +0.37, *p*-value = 0.003) and the polysaccharide export protein (FC = +0.39, *p*-value = 0.007) were significantly downregulated at 41 °C. Furthermore, the induction of the *dnaK* *chaperone* was validated at 38 °C (FC = +2.0, *p*-value = 0.02), reflecting an active heat-shock protein homeostasis response.

### 3.3 Temperature modulates biofilm architecture in *Cobetia marina* strain MM1IDA2H-1

**Figure 3:**
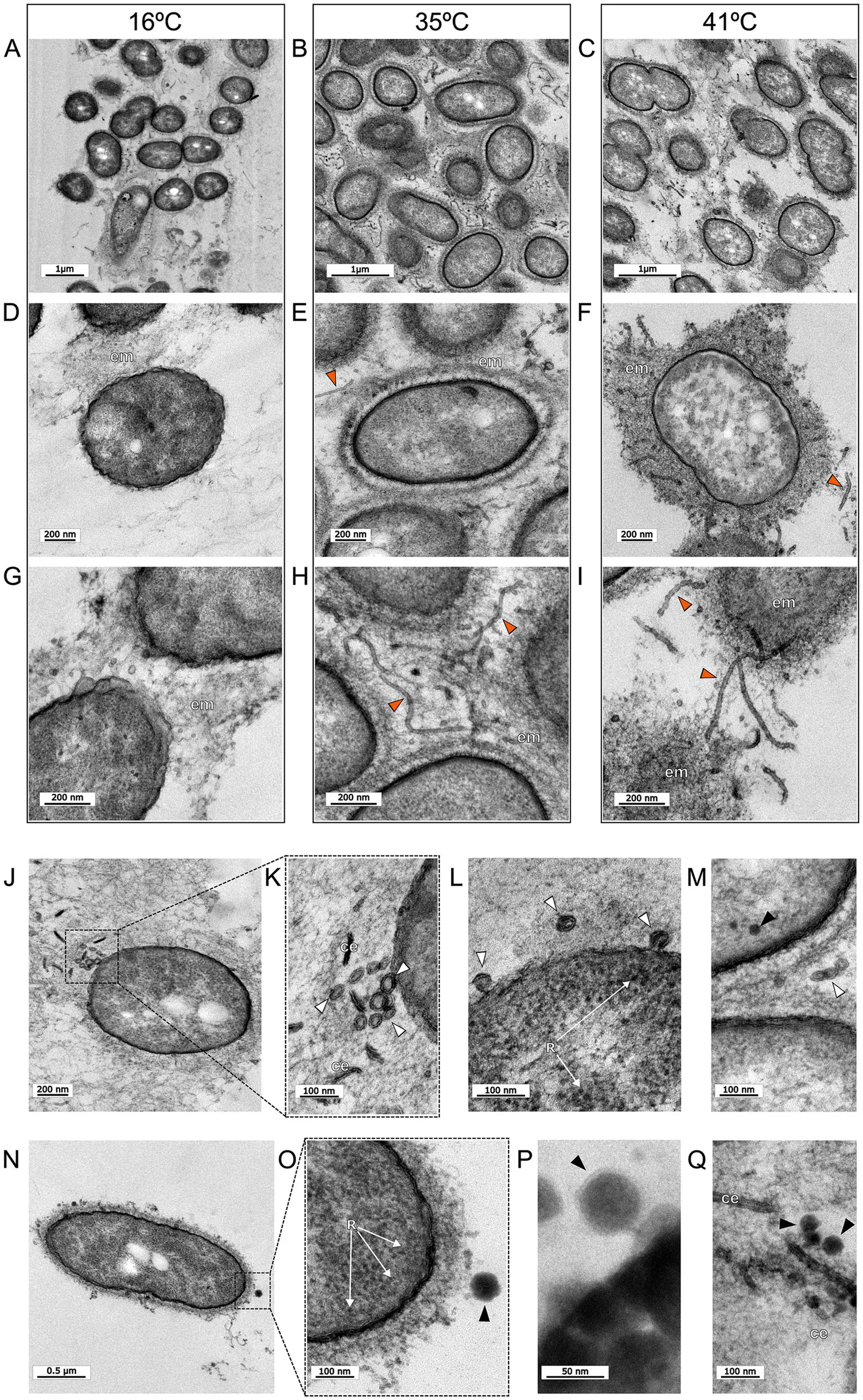
Transmission electron micrographs of the biofilm formed by *Cobetia marina* MM1IDA2H-1 under three temperature conditions (16, 35, and 41 °C). **(A–C)** Overview of the biofilm. **(D–F)** Individual bacterial cells corresponding to each condition; (em) Exopolysaccharide matrix. **(G–I)** Extracellular space. **(J, K)** High-magnification views of bacterial ultrastructure at 41 °C. **(L)** Bacterial ultrastructure at 16 °C. **(M–Q)** Bacterial ultrastructure at 35 °C. R, electron-dense granules compatible with ribosomes; (ce) Gram-negative cell envelope. The white arrowhead indicates structures morphologically consistent with outer membrane vesicles (OMVs). Orange arrowheads indicate extracellular fibrillar structures consistent with curli-like fibers. Black arrowheads indicate bacteriophage-like particles.

To investigate the effect of temperature on social behavior and biofilm formation in *C. marina* strain MM1IDA2H-1, biofilm ultrastructure was examined using transmission electron microscopy (TEM) and confocal microscopy at 16 °C, 35 °C, and 41 °C. The results revealed that temperature modulates the structure of the biofilm. As shown in Figures 3A, a uniform extracellular matrix can be observed around bacterial cells at 16 °C, resulting in low electron contrast. At 35 °C, the extracellular matrix is heterogeneous, comprising diverse structural elements. At 41 °C, *C. marina* strain MM1IDA2H-1 forms a capsule (Figure 3C). The extracellular matrix contained structures morphologically consistent with outer membrane vesicles (OMVs) (Kulp and Kuehn, 2010; Charpentier et al., 2023), electron-dense granules distributed throughout the cytoplasm compatible with ribosomes (Pilhofer et al., 2010; Cushnie et al., 2016; Mehdiyeva et al., 2023) bacteriophage-like particles associated with the bacterial cell envelope, filamentous structures consistent with pili-, fimbriae-, or flagella-like appendages, as well as extracellular fibrillar assemblies consistent with amyloid-like material (Figures 3J–Q). Under 35 °C, bacteriophage-like particles appeared attached to the outer membrane (Figure 3P). These observations suggest that temperature influences biofilm bacterial viability and composition, with fewer biofilm-associated cells under thermal stress. To macroscopically validate the structural differences observed, Congo Red staining of curli-like structures in *C. marina* strain MM1IDA2H-1 colonies grown at different temperatures was performed (Supplementary Figure S4). At 16 °C and 35 °C, *C. marina* strain MM1IDA2H-1 exhibited smooth, glossy, and pale/white colony morphotypes. This visual profile strongly aligns with our TEM observations, confirming that the thick, highly hydrated biofilm formed at lower temperatures is predominantly composed of exopolysaccharides (EPS) with low affinity for Congo Red. Conversely, incubation above the optimum temperature (38 °C and 41 °C) triggered a pronounced morphological transition, increasing red pigmentation and dense peripheral spotting with temperature. This distinctive phenotype is consistent with amyloid-like curli fibers that bind Congo Red in mature, plate-grown colonies during stationary phase.

**Figure 4:**
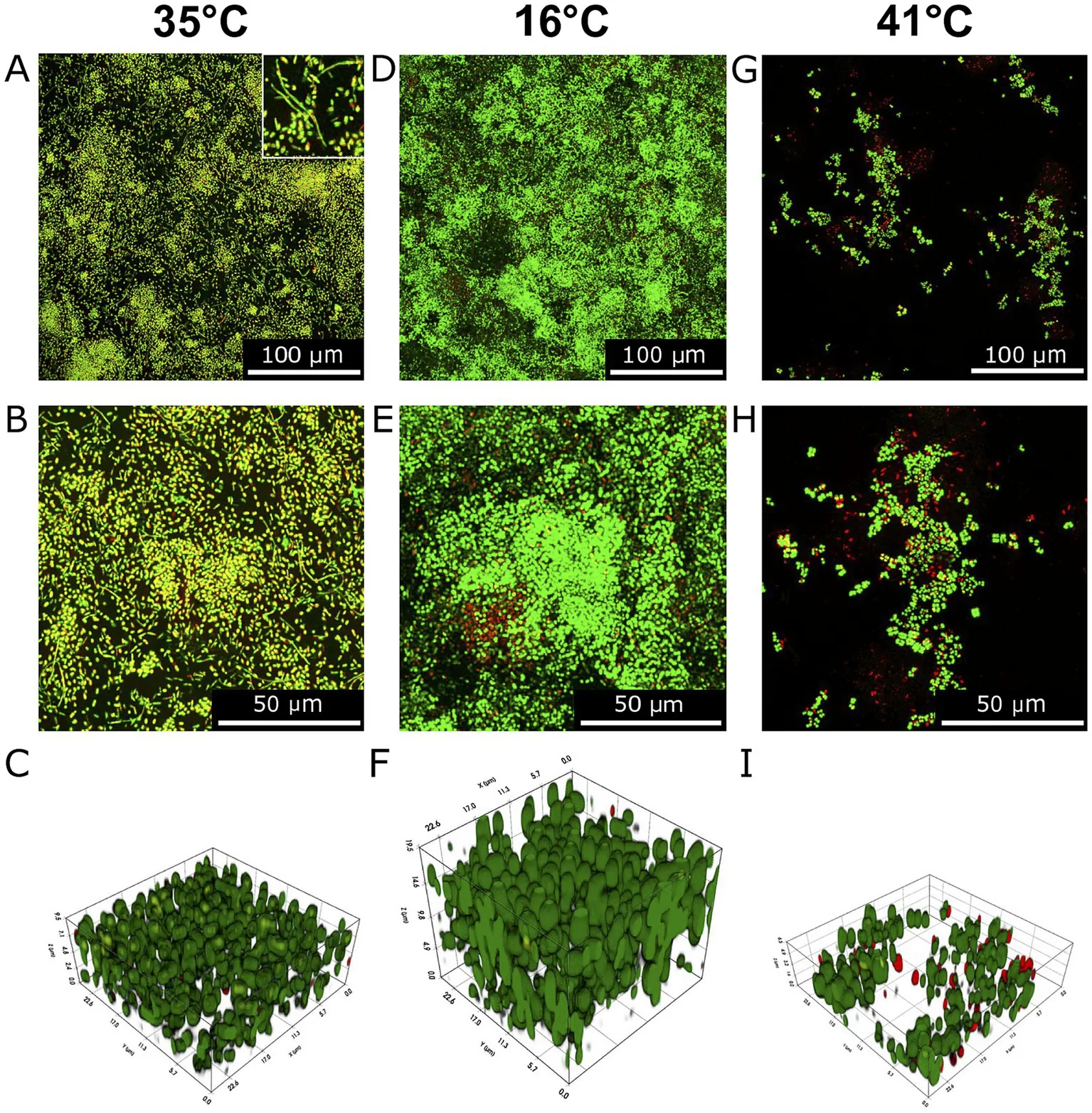
Confocal microscopy and three-dimensional reconstructions of *Cobetia marina* MM1IDA2H-1 biofilm at different temperatures. **(A–C)**: Representative merged maximum-intensity projections **(A)**, regions of interest (ROIs) acquired at higher magnification (2.5x zoom) **(B)**, showing biofilm structural details and representative three-dimensional reconstructions of central regions **(C)** illustrating overall biofilm architecture from ROIs of *C. marina* biofilms formed at 35 °C. Filamentous bacterial morphologies were observed exclusively at 35 °C (A, inset). **(D–F)**: Representative merged maximum-intensity projections **(D)**, regions of interest (ROIs) acquired at higher magnification (2.5x zoom) **(E)**, showing biofilm structural details and representative three-dimensional reconstructions of central regions **(F)** illustrating overall biofilm architecture from ROIs of *C. marina* biofilms formed at 16 °C. **(G–I)**: Representative merged maximum-intensity projections **(G)**, regions of interest (ROIs) acquired at higher magnification (2.5x zoom) **(H)**, showing biofilm structural details and representative three-dimensional reconstructions of central regions **(I)** illustrating overall biofilm architecture from ROIs of *C. marina* biofilms formed–at 41 °C. All imag–es show SYTO 9 (green) and propi dium iodide (red) staining.

The structural characteristics of *C. marina* strain MM1IDA2H-1 biofilms were further analyzed using viability staining, confocal laser microscopy, and three-dimensional reconstruction (Figure 4). At 35 °C (Figures 4A–C), the optimal growth temperature for *C. marina* strain MM1IDA2H-1, biofilms exhibited a mean thickness of 7.95 ± 0.37 μm, with a mean bacterial volume of 9,018 ± 2,361 μm^3^, occupying 8.43 ± 2.35% of the total ROI volume. Bacteria with compromised cell membranes, suggestive of cell death, were identified using propidium iodide staining and accounted for 1,549 ± 378 μm^3^, representing 17.2 ± 1.45% of the total bacterial volume. Filamentous bacteria were observed exclusively under this condition (Figure 4A, inset). At 16 °C (Figures 4D–F), the biofilm exhibited the greatest thickness among all conditions (16.8 ± 1.64 μm, *p-*value < 0.001), along with the highest total bacterial volume (14,532 ± 2,591 μm^3^, *p*-value <0.001). However, biofilm occupancy of the ROI volume was not significantly different between 16 °C and 35 °C (6.43 ± 0.91%, *p*-value = 0.072). The total volume of putative dead bacteria (132 ± 37.7 μm^3^, *p*-value <0.001) and their proportion relative to total bacterial volume (0.93 ± 0.26%, *p*-value <0.001) were significantly lower compared to the 35 °C condition. At 41 °C (Figures 4G–I), the biofilm displayed the least thickness (5.00 ± 0.53 μm, *p-*value <0.001), total bacterial volume (1,623 ± 566 μm^3^, *p-*value <0.001), and ROI occupancy (2.39 ± 0.69%, *p*-value <0.001) among the tested conditions. The total volume of putative dead bacteria was lower than at 35 °C (191 ± 90.3 μm^3^, *p*-value <0.001) but did not differ significantly from that at 16 °C (*p-*value = 0.19). The proportion of bacteria with compromised cell membrane relative to total bacterial volume was also lower than at 35 °C, reaching 11.7 ± 4.25% (*p*-value = 0.03).

### 3.4 Adaptive laboratory evolution (ALE) reveals thermal tolerance limits and biofilm trade-offs in *Cobetia marina* strain MM1IDA2H-1

**Figure 5:**
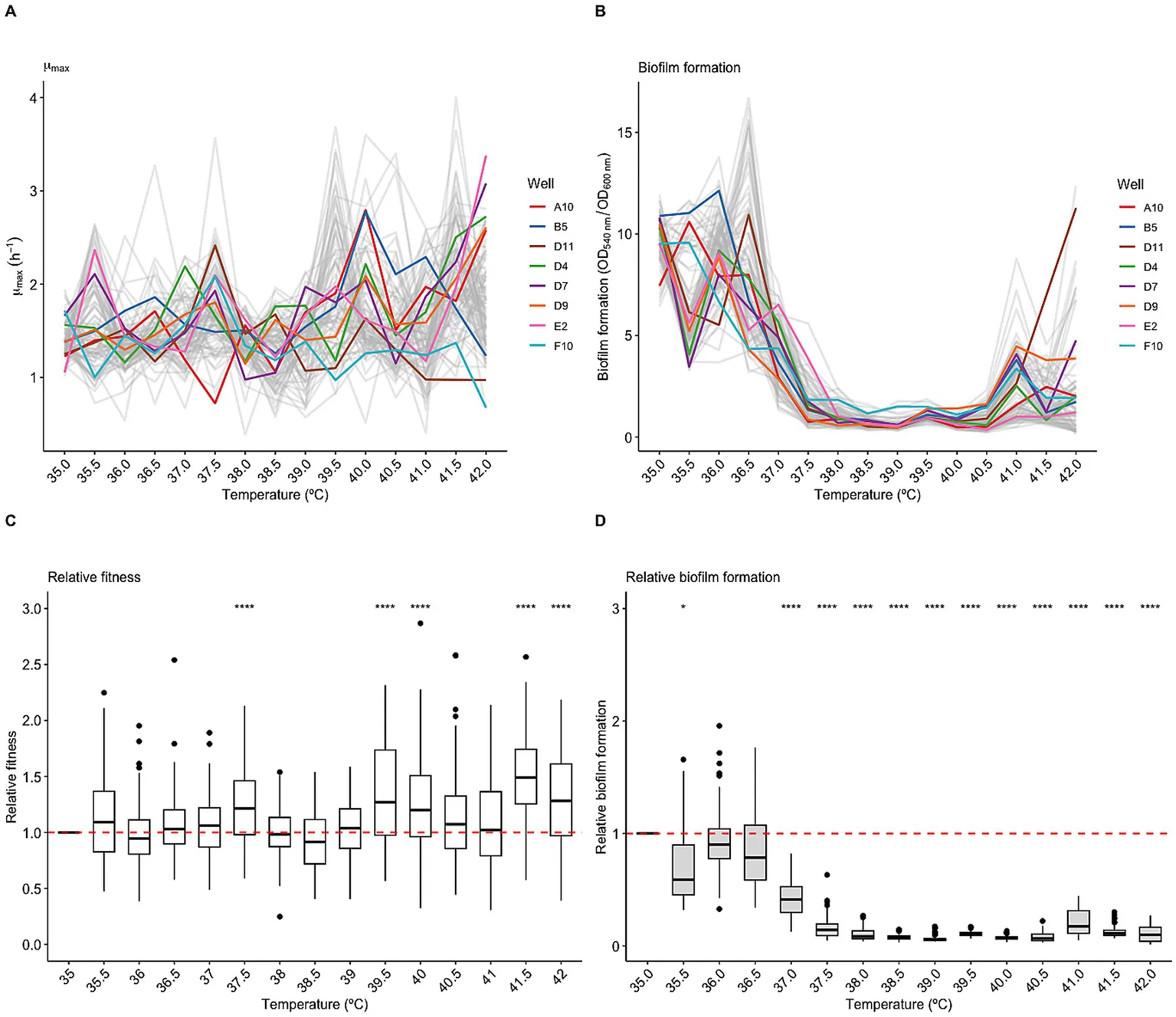
Evolution of *Cobetia marina* MM1IDA2H-1 growth rate and biofilm formation at increasing temperatures. **(A)** Evolution of growth rates of *C. marina*-derivatives at different temperatures. **(B)** Evolution of biofilm of *C. marina*-derivatives across the evolutionary process. **(C)** Relative fitness of *C. marina*-derivatives at different temperatures respect 35 °C. **(D)** Relative biofilm formation of *C. marina*-derivatives at different temperatures with respect to 35 °C. Overall differences among temperatures were assessed using the Kruskal–Wallis test, followed by Dunn’s *post hoc* test with Benjamini–Hochberg correction for multiple comparisons. Asterisks indicate significant differences relative to 35 °C: *p*-value <0.05 (*), *p*-value < 0.01 (**), *p*-value <0.001 (***), and *p*-value < 0.0001 (****). Only statistically significant pairwise comparisons are shown.

To evaluate the capacity of *C. marina* strain MM1IDA2H-1 to adapt to progressive temperature increases, we subjected the strain to an adaptive laboratory evolution (ALE) experiment in Marine Broth 2216 using a 96-well plate format. The adaptive laboratory evolution (ALE) experiment spanned 14 passages (approximately 107 generations). Of the 93 initial parallel lineages, 54 independent populations reached an OD_600nm_ > 0.1 at 42.0 °C. During the evolutionary process, maximum growth rates (μ_max_) and biofilm formation capacities were determined at each thermal step to track their phenotypic trajectories (Figures 5A,B; Supplementary Figure S5).

As illustrated in Figure 5A, the maximum growth rate (μ_max_) exhibited dynamic fluctuations, characterized by four main peaks at 35.5 °C, 37.5 °C, 39.5 °C, and 41.5 °C. Concurrently, biofilm formation in most populations peaked at 36.5 °C and then declined progressively toward 38.0 °C. Between 38.0 °C and 40.5 °C, biofilm production dropped to near-undetectable levels; however, at 41.0 °C, three specific populations displayed a slight resurgence in biofilm capacity (Figure 5B).

To determine whether adaptation to progressive thermal stress incurred an evolutionary cost, we analyzed the relative fitness (μ_max_) and relative biofilm formation of the independently evolved populations across the ALE passages (Figures 5C,D). Normalization of these parameters to the optimal baseline condition (35 °C) revealed a distinct phenotypic divergence under severe thermal stress. Despite the intense thermal challenge, the evolved populations successfully maintained their relative fitness without a significant cost compared to the baseline (Figure 5C). In contrast, these same populations showed a significant reduction in relative biofilm capacity at 37 °C (*p*-value <0.001) (Figure 5D). Together, these trends illustrate a clear phenotypic trade-off across the independently evolved populations, characterized by retaining individual growth capacity at the direct expense of biofilm production as temperatures increased.

### 3.5 Genomic analysis of evolved derivatives identifies *csgD1* as a primary target of thermal selection

Following the adaptive evolution process, eight specific lineages (highlighted in Figures 5A,B) were selected for further genomic characterization based on their contrasting phenotypic trajectories regarding maximum growth rate (μ_max_) and residual biofilm formation. To identify the potential mutations driving these selected phenotypes, a single colony from each evolved population was isolated and their whole genomes were sequenced to detect variants.

The WGS analysis revealed that seven out of the eight isolated clones carried a clone-specific mutation in the locus tag BOH68_RS17530, manually curated as *csgD1*. CsgD is a LuxR-type transcriptional regulator and a master controller of biofilm formation. Across the evolved clones, three distinct mutational events were identified within this gene (Table 1), affecting different functional domains of the regulator. Specifically, a nonsynonymous substitution at residue 23 (D23Y) was observed in five strains (CM42A10, CM42D7, CM42D9, CM42E2, and CM42F10). This mutation alters the N-terminal fraction of the protein, a region essential for dimerization and phosphorylation (Zakikhany et al., 2010; Wen et al., 2017). In strain CM42D11, an insertion sequence in *csgD1* was identified, near the DNA-binding region (Wen et al., 2017), producing a frameshift. In addition to a *csgD1* substitution (L152P), strain CM42B5 exhibited a concurrent mutation in *nqrC* (F65L), a gene belonging to the *nqr* *operon* that encodes an NADH:ubiquinone oxidoreductase (Kishikawa et al., 2022). Notably, strain CM42D4 was the only isolate lacking *csgD1* mutations; instead, it carried mutations affecting the ribosomal protein bS1 (*rpsA*, Q168R) and a hypothetical protein (S151P).

**Table 1:** Characterization of the selected *Cobetia marina* MM1IDA2H-1 evolved populations and derivative clones from ALE at 42 °C.

Population	μ_max_	Max. Biomass	Biofilm formation	Strain	SNPs			
	(h^−1^)*	(OD_600 nm_)	(OD_540 nm_/OD_600nm_)		*nqrC*	*rpsA*	Hypothetical protein	*csgD1*
A10	2.59	0.108	2.009	CM42A10				D23Y
B5	1.23	0.090	ND	CM42B5	F65L			L152P
D4	2.72	0.104	2.010	CM42D4		Q168R	S151P	
D7	3.08	0.062	ND	CM42D7				D23Y
D9	2.61	0.071	ND	CM42D9				D23Y
D11	0.97	0.031	ND	CM42D11				INS 67
E2	3.38	0.138	1.228	CM42E2				D23Y
F10	0.67	0.104	1.930	CM42F10				D23Y

ND, non detected.

*μ_max_ (h^−1^) were determined using growth curves until mid-exponential phase (OD_600nm_ = 0.3).

### 3.6 In silico inference of temperature-dependent regulatory network rewiring in *Cobetia marina* strain MM1IDA2H-1

**Figure 6:**
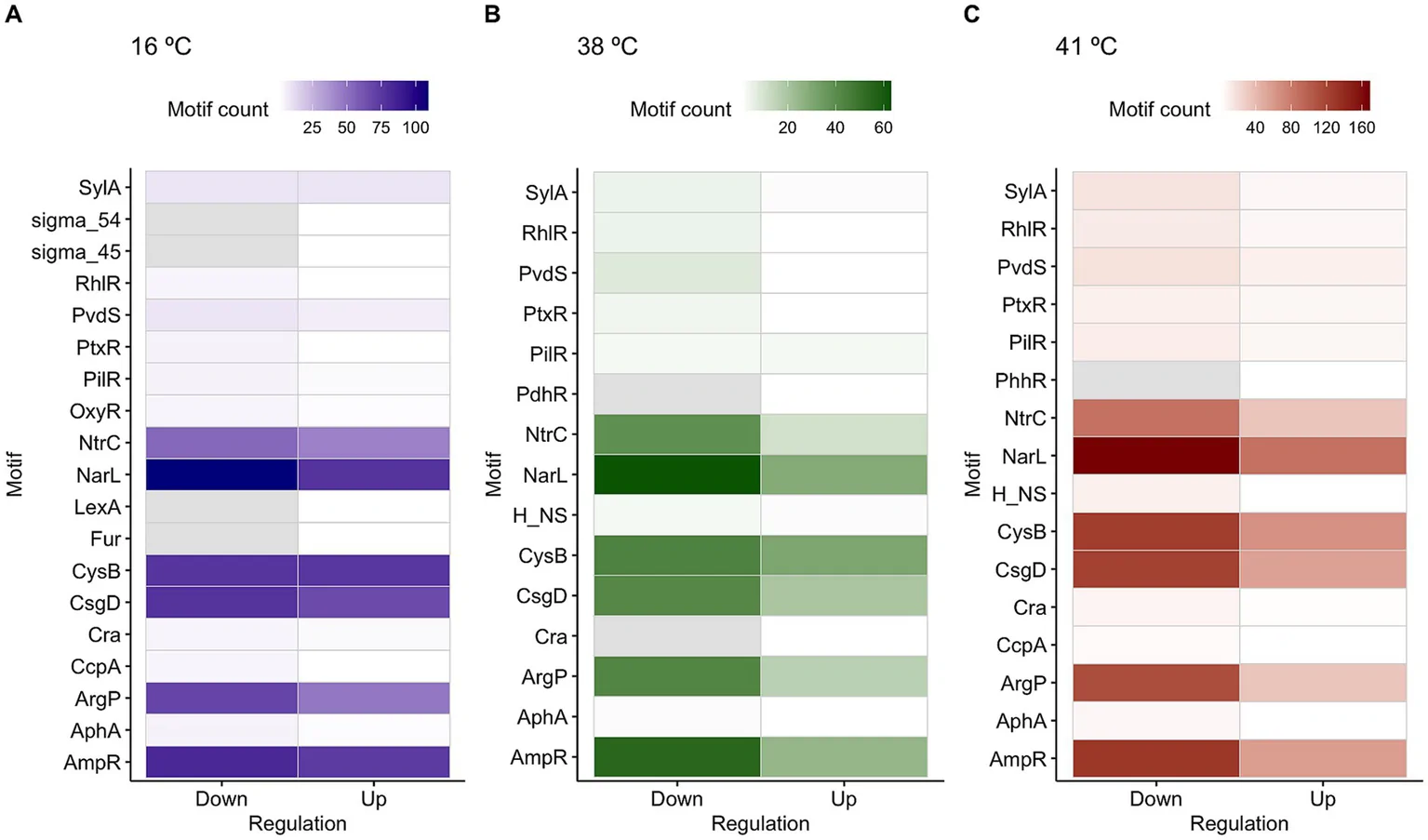
Abundance of regulatory motifs associated to differentially expressed genes in *Cobetia marina* at different temperatures with respect to 35 °C: **(A)** 16 °C; **(B)** 38 °C; **(C)** 41 °C.

To explore the potential regulatory networks involved in temperature adaptation in *C. marina* strain MM1IDA2H-1 an in silico motif-based approach was used. Transcription factor binding sites were identified within the upstream regions (350 bp) of differentially expressed genes (DEGs) at each temperature. It is important to note that computational motif detection suggests, but does not definitively prove, direct regulatory binding or causal activation/repression. Therefore, the following network reconstructions represent predicted regulatory associations that provide a hypothesis-driven framework for thermal adaptation. The regulatory motifs detected under each condition and their inferred contributions to gene up- and down-regulation are summarized in Figure 6 (Supplementary Table S6–S8).

Analysis of motif-DEG associations suggested that the putative contribution of NarL to global gene regulation increases at the extreme temperatures (16 °C and 41 °C), with the greatest number of motifs identified at 41 °C. According to these in silico predictions, the primary candidate regulators associated with temperature stress responses are NarL, NtrC, CsgD, CysB, and AmpR (Figure 6). In general, the NarL motif was frequently associated with repressed genes across all conditions (Figures 6A–C), a pattern that peaked at 41 °C, where it co-occurred with the induction of 87 genes and the repression of 169 genes. Similarly, CsgD, CysB, and AmpR motifs were predominantly associated with downregulatory profiles at all temperatures, with this effect becoming more pronounced at the highest temperature. Another predicted motif identified across all DEG sets belongs to PtxR, with six, five, and fifteen associated genes at 16 °C, 38 °C, and 41 °C, respectively. At 16 °C, four predicted motifs were uniquely detected: sigma-54, sigma-45, LexA, and Fur (associated exclusively with upregulated genes), while OxyR motifs appeared linked to both expression trends (Figure 6A). Conversely, putative H-NS motifs were solely detected in the transcriptomes of *C. marina* strain MM1IDA2H-1 growing above its optimal temperature (38 °C and 41 °C) (Figures 6B,C).

Regarding CsgD/LuxR, the in silico motif mapping revealed evidence of regulatory integration within its promoter region. The upstream region of genes encoding LuxR C-terminal-related transcriptional regulators (locus tags BOH68_RS17530, manually curated as *csgD1*, and BOH68_RS17535) contain putative binding motifs for CsgD itself, as well as for NarL and CysB. The presence of NarL, AmpR, and CysB motifs near this locus is consistent with a hypothesized upstream regulatory coupling between the global stress response and the CsgD/LuxR module. In summary, the motif-based association of DEGs infers a putative NarL-centered regulatory framework across all temperatures, complemented by auxiliary contributions from CysB, AmpR, and CsgD/LuxR, as well as the temperature-conditional participation of H-NS and PtxR.

**Figure 7:**
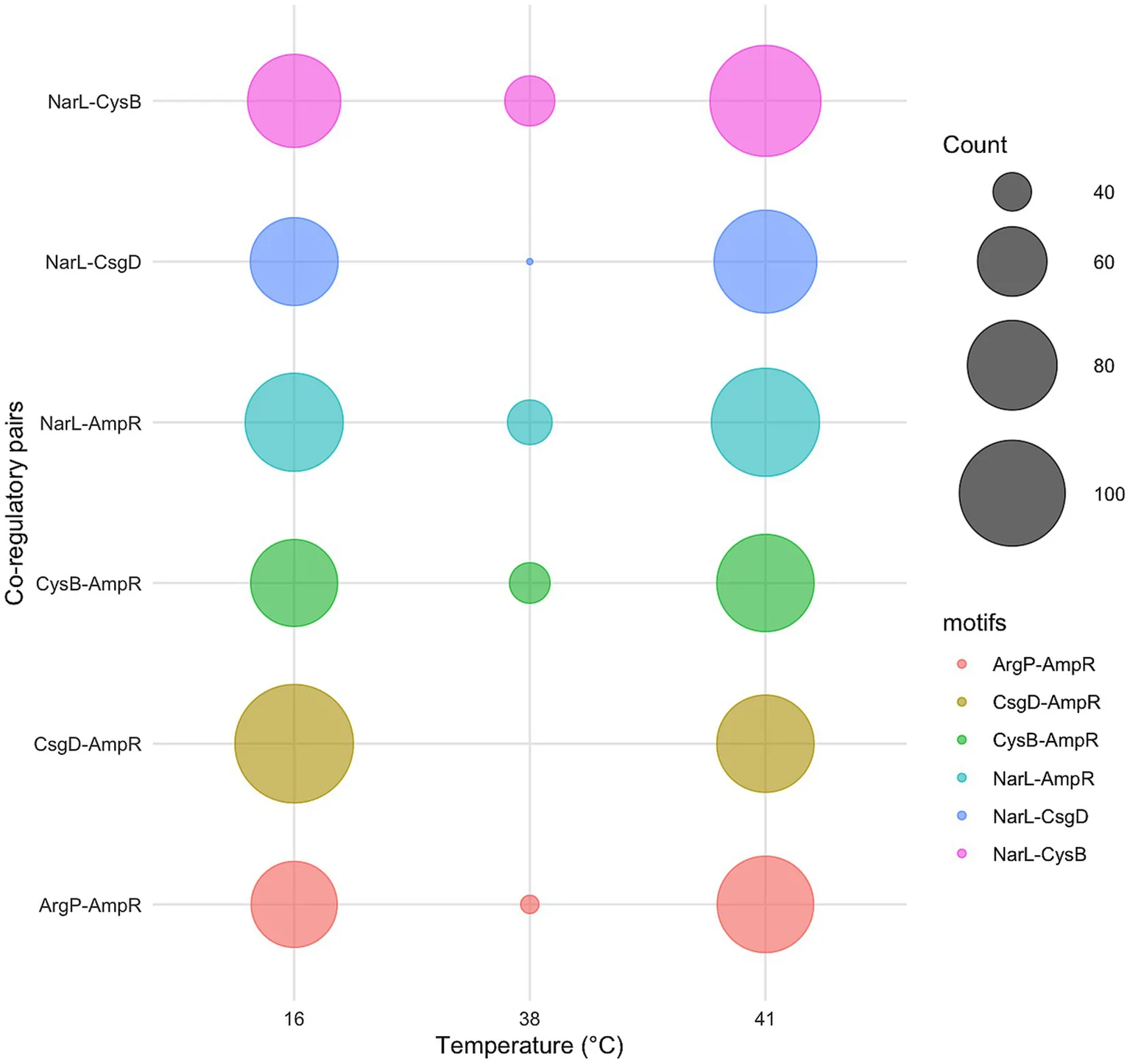
Co-regulatory dynamic and topological reprogramming across thermodynamic gradients. Co-occurrence of transcriptional regulator motifs in differentially expressed genes of *Cobetia marina* MM1IDA2H-1 at different temperatures.

Finally, to explore potential interactions among these inferred regulators, we analyzed the co-occurrence of different motifs within the same upstream regions (Figure 7; Supplementary Table S9). At the 38 °C transition boundary, the inferred network contracts notably, as evidenced by a sharp decline in the number of shared target genes across all regulatory pairs. Conversely, the transcriptomic profile at the 41 °C survival boundary is associated with a predicted expansion of co-regulatory motif occurrences. This putative stress-adaptive transcriptional state is prominently associated with the NarL–CysB and NarL–AmpR motif pairs.

## 4 Discussion

*Cobetia marina* strain MM1IDA2H-1 exhibits remarkable eurythermal condition, capable of sustaining growth, viability, and biofilm formation across a broad thermal range (Stehlik et al., 2025). To fully contextualize the response of *Cobetia marina* strain MM1IDA2H-1 to temperature, its environmental origin must be considered. This strain was originally isolated from a temperate Pacific eulittoral intertidal rock pool (Ibacache-Quiroga et al., 2013). Intertidal pools are highly dynamic, fluctuating ecosystems, often classified as “true extreme” marine habitats due to the drastic physicochemical changes that occur on both diurnal and seasonal timescales (Huggett and Griffiths, 1986; Clarke and Beaumont, 2020; Vinagre et al., 2021). Transcriptomic and structural analyses, further supported by targeted qRT-PCR validation, reveal that this adaptability is not passive but driven by highly coordinated, temperature-dependent reprogramming of ecophysiological processes. From a resource-allocation perspective, this phenotypic plasticity reflects a dynamic, optimal partitioning of metabolism to maximize cellular fitness under shifting thermodynamic regimes (Mairet et al., 2021). The substantial number of differentially expressed genes (DEGs) at 16 °C and 41 °C, in contrast to the sharply reduced number at 38 °C, indicates that 38 °C is a critical transitional boundary, while the thermal extremes represent highly specialized and distinct adaptive physiological states.

At 16 °C, the KEGG enrichment profiles and biofilm ultrastructure indicate that *C. marina* MM1IDA2H-1 prioritizes an exploratory, community-oriented state. In this context, the importance of quorum sensing in rapid biofilm formation and structural development at low temperatures has been demonstrated (Hu et al., 2017). Our transcriptomic findings, characterized by the coordinated upregulation of flagellar assembly, chemotaxis machinery, and exopolysaccharide (EPS) export pathways, support a socially cooperative and metabolically optimized strategy for surface sensing and robust biofilm development. Despite the thickest biofilm across the studied temperatures (~16.8 μm) being recorded at 16 °C, colony morphotyping via Congo Red staining and TEM ultrastructural imaging did not reveal a massive presence of amyloid-like curli fibers (Supplementary Figure S4), suggesting that the *C. marina* MM1IDA2H-1 biofilm at low temperatures is predominantly composed of exopolysaccharides—a well-documented adaptation in psychrophilic marine bacteria where extracellular polymers function as cryoprotectants (Poli et al., 2010).

Conversely, the maximum growth temperature (41 °C) triggers a protective, “bunker-like” individual survival strategy in which energetically expensive cooperative traits, such as quorum sensing, are repressed. Transcriptional resources are systematically reallocated toward essential cellular maintenance and genetic-information protection, as evidenced by the induction of DNA replication and repair systems, chaperone networks, and oxidative stress-mitigating enzymes. Extreme temperatures promote protein denaturation and unfolding, diverting biosynthetic and translational resources from active growth to chaperone-mediated stress responses and macromolecular protection (Mairet et al., 2021). At the structural level, this stress-adaptive state is associated with a flattened, minimal biofilm architecture featuring extracellular assemblies morphologically consistent with amyloid-like curli fibers (CLSM and TEM). Interestingly, while these curli-like structures are visualized in the stationary-phase biofilms (Figure 3; Figure S4), the gene expression of both *csgD1* and the curli operon is significantly repressed during the mid-exponential phase at 41 °C (Supplementary Figure S3, Table S5). This apparent temporal discrepancy is likely explained by the bacterium’s growth phase; while transcriptomic profiling was conducted during active exponential growth, the biosynthetic pathway for amyloid fimbriae is typically induced during the transition into the stationary phase (Barnhart and Chapman, 2006). A comparable physiological transition has been described in biofilms of the obligate anaerobe *Clostridium perfringens* under thermal stress, in which physical remodeling of the matrix serves as a crucial barrier that protects cells from environmental exposure (Obana et al., 2020).

When comparing growth at 38 °C and 35 °C, the transient growth arrest observed may be associated with the selective downregulation of nonessential biosynthetic and motility-related pathways. Repression of flagellar, chemotaxis, cell-wall remodeling, and pilus-assembly genes indicates the reallocation of energetic resources from motility and dynamic adhesion to heat-shock and stress-adaptive processes. Additionally, the observed downregulation of a DNA cytosine methyltransferase (BOH68_RS11970, LFC ≈ −1.03) suggests that epigenetic regulatory mechanisms may participate in the response to elevated temperature (Sharaf et al., 2026). Although the functional consequences remain to be experimentally validated, altered methyltransferase expression could potentially influence genome-wide methylation patterns and thereby contribute to the transcriptional reprogramming observed under heat stress. The combined repression of energetically expensive functions and the putative modulation of epigenetic regulatory mechanisms suggest a coordinated shift in cellular priorities under thermal stress. Together, these findings support a coordinated “stop-and-reprogram” strategy underlying the diauxic, sigmoidal growth kinetics observed at 38 °C (Dinamarca et al., 2025). This physiological restructuring at the 38 °C boundary illustrates the cellular manifestation of asymmetric evolutionary constraints, where transitioning between distinct thermal niches requires a temporary suspension of growth to overcome thermodynamic barriers before adaptation can proceed (Roemhild et al., 2015). Macroscopically, our Congo Red assay (Supplementary Figure S4) supports this transitional state: colonies at 38 °C exhibit an intermediate (light red) phenotype, suggesting moderate deposition of extracellular assemblies morphologically consistent with amyloid-like curli fibers.

To translate environmental temperature conditions into these coordinated phenotypic outputs, *C. marina* MM1IDA2H-1 requires a highly responsive gene-regulatory architecture. Our in-silico motif-based reconstruction suggests a putative temperature-dependent regulatory landscape sustained by a predicted core comprising the response regulators NarL and NtrC, the LysR-type regulator CysB, and the biofilm master controller CsgD. These regulators collectively integrate nitrate sensing, nitrogen metabolism, sulfur homeostasis, and multicellular surface-associated behaviors (Hengge, 2020; Kompaniiets et al., 2024; Mayo-Pérez et al., 2024), suggesting a regulatory architecture that couples environmental stress perception with metabolic reprogramming and biofilm development. Consistent with this hypothesis, the in silico motif analysis identified putative binding sites for NarL, CysB, and CsgD itself within the upstream regulatory region of *csgD1*, suggesting potential autoregulatory and environmentally responsive control of this putative biofilm regulatory module. In uropathogenic *E. coli,* the nitrate-responsive regulator NarL modulates *csgD* expression, linking respiratory adaptation to biofilm development (Martín-Rodríguez et al., 2020).

Consequently, the predicted NarL–NtrC–CysB–CsgD regulatory core identified in *C. marina* strain MM1IDA2H-1 may represent an integrative network through which temperature-induced physiological fluctuations are translated into coordinated shifts in growth rate modulation, energy allocation, metabolic adaptation, and biofilm-forming capacity. Given the eurythermal nature of *C. marina*, this regulatory core—which links nutrient sensing, growth rate control, cellular respiration, electron acceptor availability, osmolarity, and structural integrity—is highly consistent with the master role of temperature in marine environments, where it interactively modulates each of these physiological variables (Adams and Russell, 1992).

In addition to the transcriptomic response of *C. marina* MM1IDA2H-1 to temperature according to its eurythermal condition, this study aimed to determine whether genetic modifications actively complement and expand *C. marina*’s adaptive capabilities under sustained heat stress. The resulting ALE landscape revealed that populations reaching the thermal boundary at 42 °C retained relative growth fitness despite severe heat stress, whereas biofilm production progressively collapsed with increasing temperature. While the transfer inoculum (1 μL, ≈10 ^5^–10 ^6^ cells) used during the ALE passages establishes a broad bottleneck designed to minimize random genetic drift, we cannot definitively rule out the influence of founder effects without deep population sequencing. However, the striking genetic parallelism of *csgD* *mutations* across seven independent lineages, combined with the transcriptomic repression of biofilm pathways, strongly supports a selection-driven adaptive response. By mutating this key regulator, *C. marina* strain MM1IDA2H-1seems to hardwire a stress-adaptive state, permanently resolving the metabolic trade-off to ensure survival and growth in near-lethal thermal environments.

Three non-synonymous mutations were identified among clones across different regions of the gene, two of which were in the N-terminal region. Mutations in position 23 are contiguous to a key region (residue 21/22) for dimerization of the protein (Wen et al., 2017), and where mutations have proven to affect biofilm formation. Genetic modification in this fraction has been shown to alter the regulator’s activity and bacterial phenotypes, including biofilm formation. Despite five clones sharing the D23Y variant, their biofilm phenotypes differed. *csgD* *gene expression* has been proven to vary among clonal populations (Grantcharova et al., 2010). These results suggest that, rather than continually expending energy to transiently repress the biofilm circuit via global regulators, an evolutionary pathway emerges that alters the *csgD1* *master switch*. The persistence of residual biofilm formation at high temperatures suggests that these mutations do not act as an absolute “ON/OFF” knockout but rather as a regulatory “dimmer” that downregulates matrix overproduction to a variable and sustainable level. This agrees with our CLSM, TEM, and Congo Red staining results, which show reduced, but not absent, capacity to form biofilms at high temperature (41 °C), with the presence of curli amyloids, suggesting that these structures may play a role in biofilm thermal resistance.

Finally, our findings regarding the thermal adaptability of *Cobetia marina* align with and extend established physiological models for other ubiquitous marine Pseudomonadota, including well-studied halophilic *Vibrio* species, where the interactive effects of salinity and temperature govern membrane lipid composition and cellular boundary integrity, thereby preserving homeostatic function (Sheikh et al., 2022). In *C. marina* strain MM1IDA2H-1, this environmental integration is similarly critical. While our strain maintains stable growth kinetics across a broad thermal range, severe thermal upshifts (above 38 °C) induce a distinct transition in which the bacterium downregulates energy transport and outer membrane proteins, such as TonB-dependent receptors, while activating DNA repair and capsular structures. This physiological restructuring reflects a broader ecological strategy discussed by Ore (Oren, 2024), in which halophilic microorganisms in highly dynamic marine and hypersaline niches deploy specific osmotic and thermoprotective mechanisms to sustain biochemical functioning. As shown by Brumfield Kyle et al. (2023), physical factors such as temperature and salinity serve as key environmental drivers governing the abundance, lifestyle transitions, and ecological occurrence of these marine bacteria in coastal waters. In this context, studying *C. marina’s* coordinated physiological “stop-and-reprogram” strategy helps identify the molecular and cellular mechanisms that enable eurythermal bacteria to translate environmental temperature fluctuations into defined ecological lifestyles.

The multidimensional approach of this study, integrating transcriptomics, cell structures, three-dimensional spatial biofilm profiling, and an adaptive laboratory evolution (ALE) experiment coupled with genome sequencing, unveils strategies based on gene-regulatory networks (GRNs) that govern the ecological success of the widespread, eurythermal marine bacterium *Cobetia marina*. Rather than displaying a passive tolerance to temperature variation for growth, *Cobetia marina* exhibits an active information-processing capability that dynamically translates physical stimuli into coordinated physiological and behavioral transitions. This sophisticated regulatory plasticity highlights the mechanisms that enable ubiquitous Pseudomonadota to maintain functional viability across the highly dynamic oceans.

## Data Availability

The datasets presented in this study can be found in online repositories. The names of the repository/repositories and accession number(s) can be found in the article/Supplementary material.

## Footnotes

1
https://www.kegg.jp/blastkoala/

2
www.prodoric.de
