# Supplementary material for: Thermal modulation of transcriptional states, biofilm architecture and survival strategies in Cobetia marina
Source: Front Microbiol. 2026 Sep 9;17:1858040. doi: 10.3389/fmicb.2026.1858040 (PMC13597858; doi:10.3389/fmicb.2026.1858040)
Supplement: Supplementary file 1 [file Data_Sheet_1.zip › supplemetary_material/Supplementary_Figure_S5.pdf]

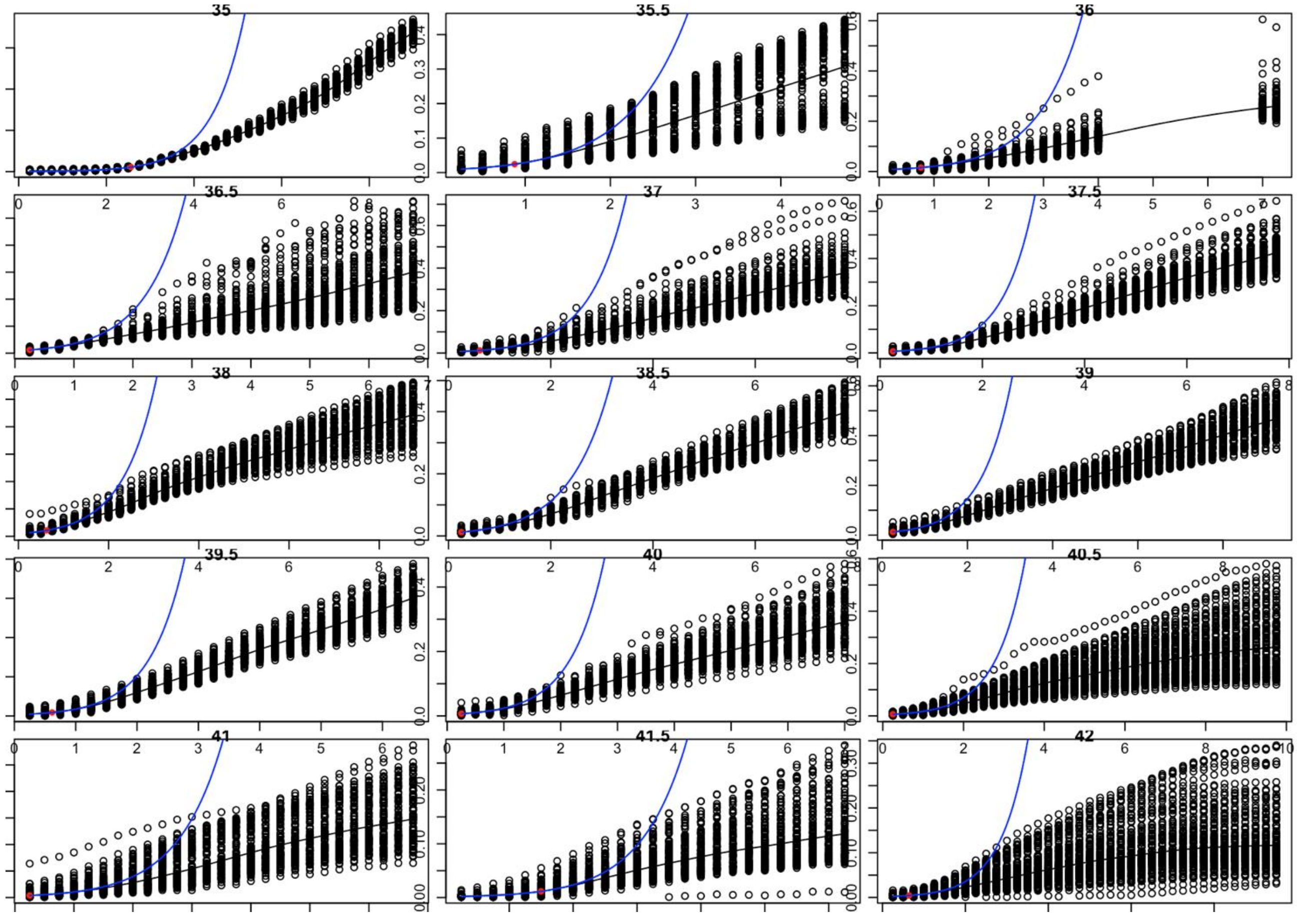

Supplementary Figure S4. Growth curves modeling of *C. marina* evolving populations at different temperaturas.
