# Supplementary material for: Thermal modulation of transcriptional states, biofilm architecture and survival strategies in Cobetia marina
Source: Front Microbiol. 2026 Sep 9;17:1858040. doi: 10.3389/fmicb.2026.1858040 (PMC13597858; doi:10.3389/fmicb.2026.1858040)
Supplement: Supplementary file 1 [file Data_Sheet_1.zip › supplemetary_material/Supplementary_Figure_S4.pdf]

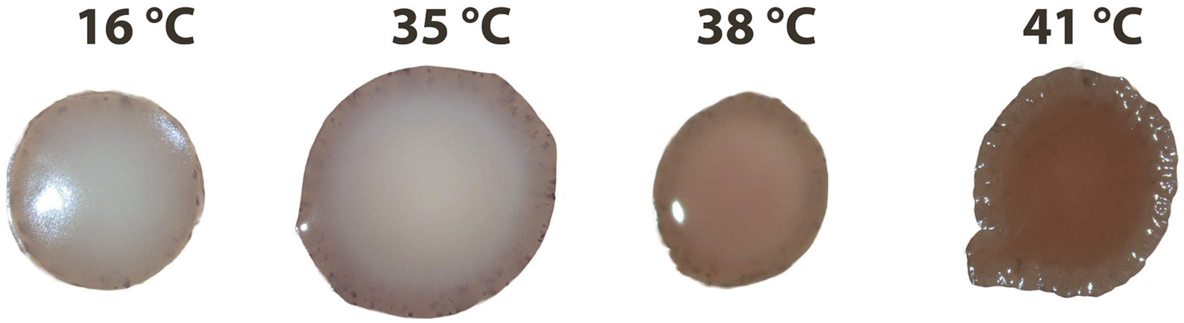

Supplementary Figure S4. Colony morphotyping of *Cobetia marina* MM1IDA2H-1 via Congo red staining across a thermal gradient. Marine agar plates supplemented with Congo red were incubated at 16 °C **(A),** 35 °C **(B)**, 38 °C **(C)**, and 41 °C **(D)** for 48–72 h. Colonies grown at 16 °C and 35 °C exhibited a smooth, pale phenotype (indicating low dye affinity), whereas those incubated at 38 °C and 41 °C displayed a distinctive, highly pigmented red morphotype, compatible with the deposition of amyloid-like curli fibers
