# Supplementary material for: Thermal modulation of transcriptional states, biofilm architecture and survival strategies in Cobetia marina
Source: Front Microbiol. 2026 Sep 9;17:1858040. doi: 10.3389/fmicb.2026.1858040 (PMC13597858; doi:10.3389/fmicb.2026.1858040)
Supplement: Supplementary file 1 [file Data_Sheet_1.zip › supplemetary_material/Supplementary_Figure_S1.pdf]

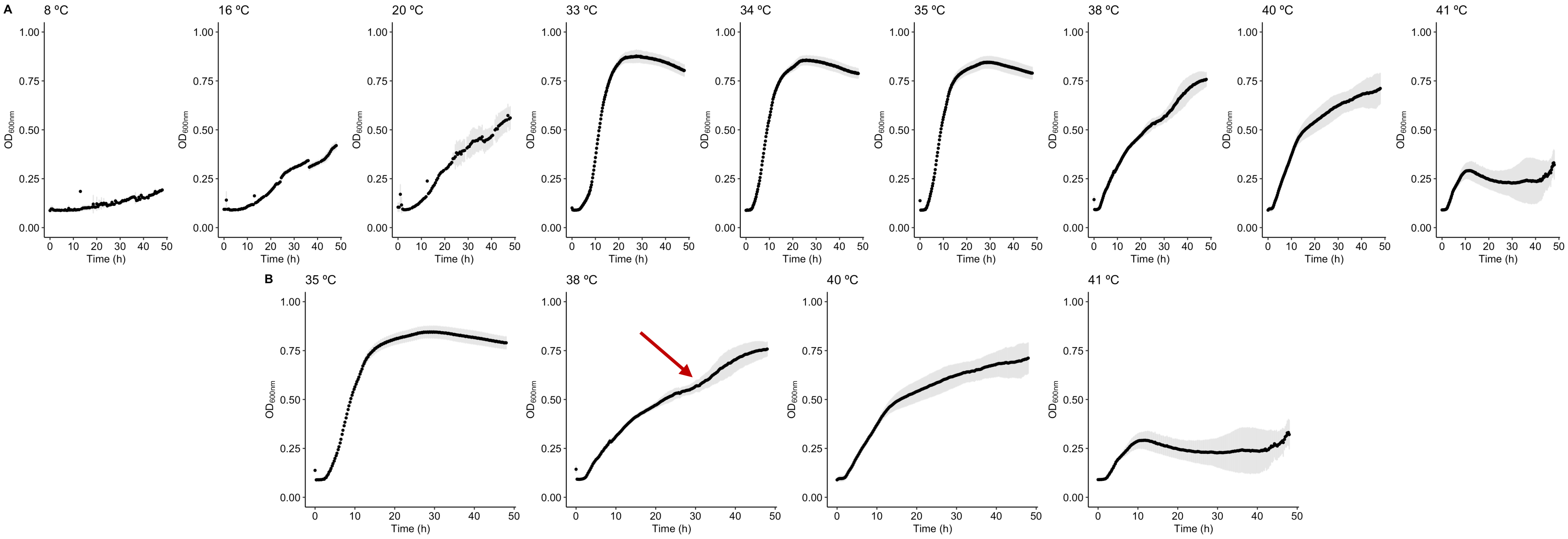

Supplementary Figure S1. Growth of *Cobetia marina* MM1IDA2H-1 at different temperatures. A: Growth curves of *C. marina* at 8 °C - 41 °C. B: Close up look of the growth curves at high temperatures.
