# Supplementary material for: Thermal modulation of transcriptional states, biofilm architecture and survival strategies in Cobetia marina
Source: Front Microbiol. 2026 Sep 9;17:1858040. doi: 10.3389/fmicb.2026.1858040 (PMC13597858; doi:10.3389/fmicb.2026.1858040)
Supplement: Supplementary file 1 [file Data_Sheet_1.zip › supplemetary_material/Supplementary_Figure_S3.pdf]

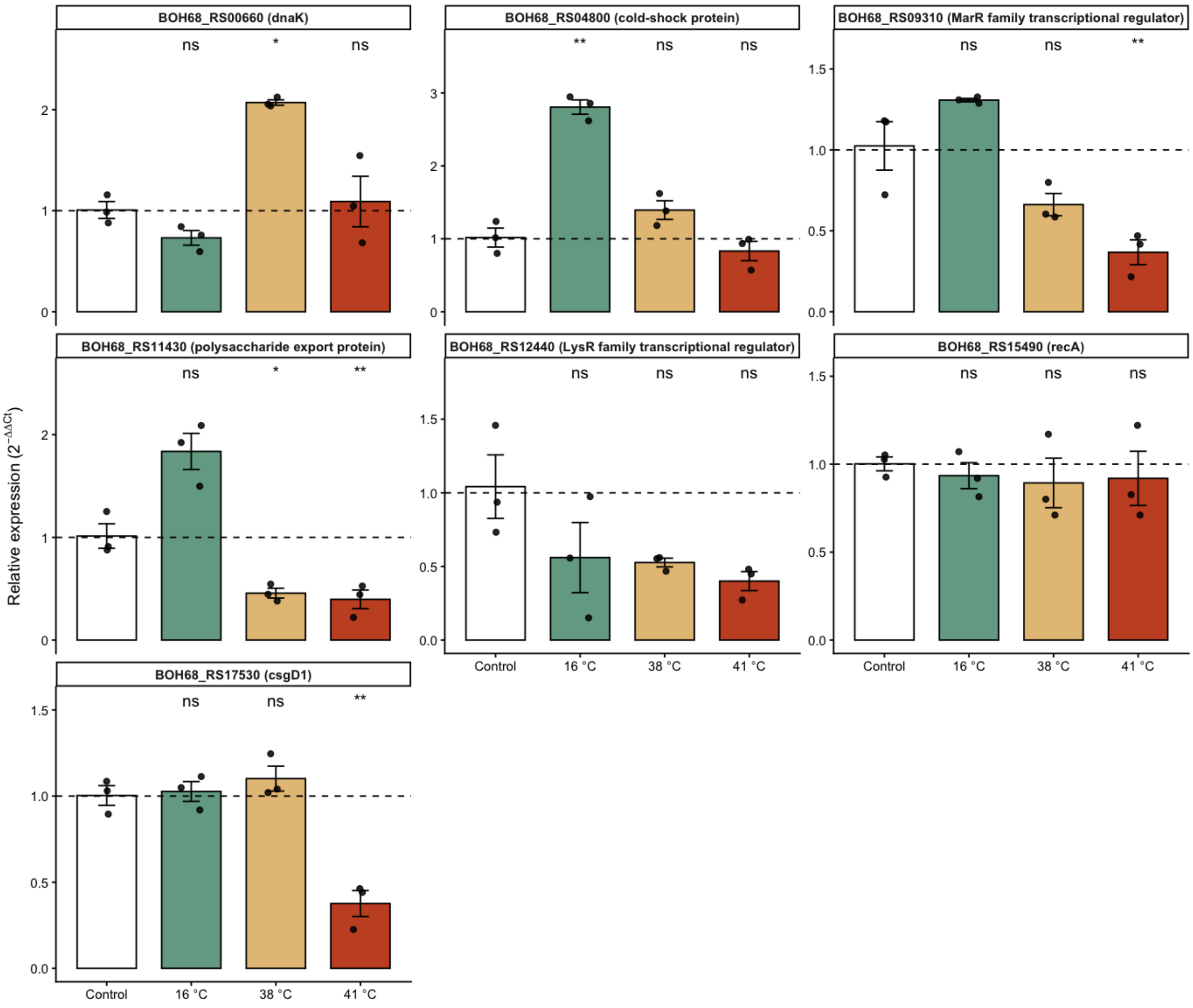

Supplementary Figure S3. Relative gene expression of selected genes determined by qRT–PCR. Under different temperature conditions. Expression levels at 16 °C, 38 °C, and 41 °C were quantified and normalized to the reference gene rpoD. Relative expression was calculated using the $2^{-}\Delta\Delta Ct$ method, with the control condition (35 °C) set to 1. Bars represent mean fold change (FC) ± standard error of the mean (SEM) from three biological replicates, with individual data points shown. Statistical significance relative to the control was determined using linear models followed by Dunnett's test ($p \leq 0.05$, $p \leq 0.01$, $p \leq 0.001$; ns, not significant).
