# Supplementary material for: Thermal modulation of transcriptional states, biofilm architecture and survival strategies in Cobetia marina
Source: Front Microbiol. 2026 Sep 9;17:1858040. doi: 10.3389/fmicb.2026.1858040 (PMC13597858; doi:10.3389/fmicb.2026.1858040)
Supplement: Supplementary file 1 [file Data_Sheet_1.zip › supplemetary_material/Supplementary_Figure_S2.pdf]

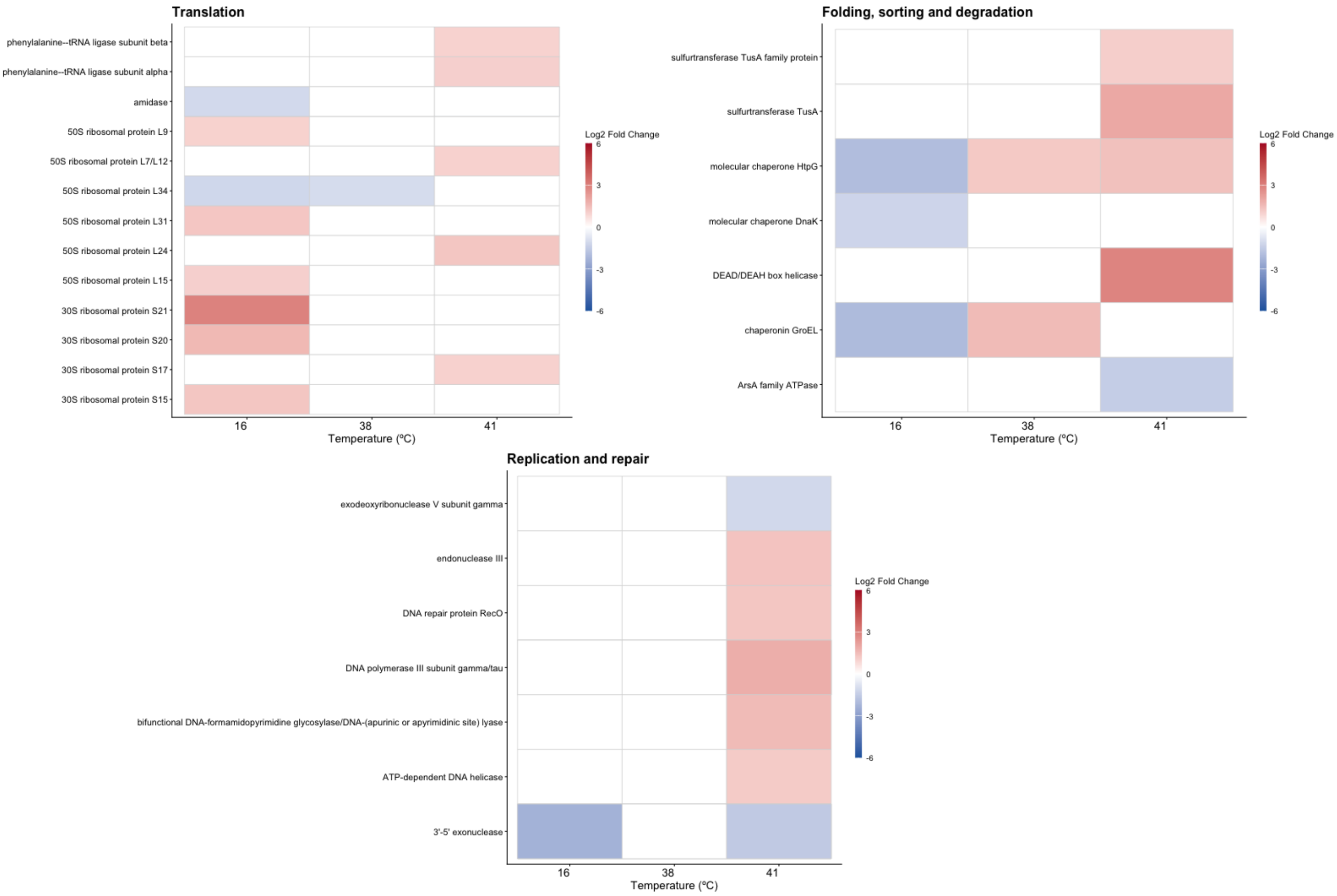

Supplementary Figure S2. Differencial gene expression at different temperatures. Differential genes expression of genes belonging to the following KEGG modules: Translation; Folding, sorting and degradation; and, Replication and repair.
