# Supplementary material for: Thermal modulation of transcriptional states, biofilm architecture and survival strategies in Cobetia marina
Source: Front Microbiol. 2026 Sep 9;17:1858040. doi: 10.3389/fmicb.2026.1858040 (PMC13597858; doi:10.3389/fmicb.2026.1858040)
Supplement: Supplementary file 1 [file Data_Sheet_1.zip › supplemetary_material/Supplementary_Table_S2.pdf]

Supplementary Table S2. Genes and primer sequences

Locustag	Annotation	Primer forward (5´- 3´)	Primer reverse (5´- 3´)
BOH68_RS15200	*rpoD*	ACACGGTTCAGCTTGTTGAT	CCGTCGTGGTTACAAGTTCT
BOH68_RS15490	*recA*	TGTTCTTCACGACCTTGACG	GGTAACGCGCTGAAGTTCTA
BOH68_RS04800	Cold-shock protein	TTGATGTTTGCGGCCTGAA	TCGCGCACTTCTCTGAAATT
BOH68_RS12440	LysR family transcriptional regulator	AGCAATATCAGTGGCAGCTT	AGGATGATCTGCTGTCCTCT
BOH68_RS11430	Polysaccharide export protein	CCTGGATTTCGGTGCTGTAG	CGCAGGATGTCGTCTATGTC
BOH68_RS09310	MarR family transcriptional regulator	CACTGATGACACTCCCGAAG	CAGACGGTTGAGGCGATAG
BOH68_RS17530	*csgD1*	GCTATCTCGGTATTGGAGGC	CTTGCTGGACTTCTACCGTC
