# Supplementary material for: Thermal modulation of transcriptional states, biofilm architecture and survival strategies in Cobetia marina
Source: Front Microbiol. 2026 Sep 9;17:1858040. doi: 10.3389/fmicb.2026.1858040 (PMC13597858; doi:10.3389/fmicb.2026.1858040)
Supplement: Supplementary file 1 [file Data_Sheet_1.zip › supplemetary_material/Supplementary_Table_S1.pdf]

Supplementary Table S1. RNA and sequencing quality control

Sample	RIN	Total Read Pairs	Total Reads (R1 + R2)	Total bp > Q30	% bp > Q30	Mapping Rate (%)
T16R1	8.4	21399397	42798794	5466898370	93,717	98.31
T16R2	8.0	19266822	38533644	4899361375	93,734	98.27
T16R3	8.1	20054204	40108408	5079618408	93,854	98.28
T35R1	8.7	20727639	41455278	5323426271	93,569	98.40
T35R2	6.5	27248118	54496236	6950610644	93,523	98.40
T35R3	8.8	29553029	59106058	7491795895	93,516	98.32
T38R1	7.8	21896486	43792972	5626097954	93,109	98.20
T38R2	8.7	25471250	50942500	6504066119	93,387	98.18
T38R3	8.7	23239326	46478652	5991787259	93,089	98.01
T41R1	8.4	23200544	46401088	5942211326	93,242	98.22
T41R2	8.3	26068167	52136334	6665597582	92,848	98.06
T41R3	7.9	18734591	37469182	4799966982	93,108	98.11
